# Does Financial Literacy Affect Household Financial Behavior? The Role of Limited Attention

**DOI:** 10.3389/fpsyg.2022.906153

**Published:** 2022-06-20

**Authors:** Shulin Xu, Zhen Yang, Syed Tauseef Ali, Yunfeng Li, Jingwen Cui

**Affiliations:** ^1^School of Economics, Guangzhou College of Commerce, Guangzhou, China; ^2^School of Business Administration, Dongbei University of Finance and Economics, Dalian, China; ^3^School of Accounting, Dongbei University of Finance and Economics, Dalian, China; ^4^College of Finance, Jiangxi Normal University, Nanchang, China; ^5^College of Political Science and Law, Jiangxi Normal University, Nanchang, China

**Keywords:** financial literacy, limited attention, financial behavior, mechanism analysis, financial information

## Abstract

Financial literacy is essential for every individual concerned with public welfare and household portfolio choices. In this study, we investigate the impact of household financial literacy on individuals’ financial behavior using the China Household Financial Survey Data (CHFS) of 2015 and 2017. The results show that financial knowledge has significant current, long-term, and dynamic effects on financial behavior. This finding suggests that financial literacy is an important factor in shaping and improving financial behavior. Second, financial literacy can improve residents’ limited attention, and residents with high attention tend to have formal bank accounts, participate in the stock market, and engage in financial behaviors in situations such as risky financial markets. High attention also helps to improve residents’ financial behavior. This relationship suggests that financial literacy positively impacts formal bank account holding, participation in financial markets, participation in commercial insurance, participation in pension plans, and credit card holdings through limited attention channels that facilitate access to specific financial information. In addition, heterogeneity analysis showed that the impact of financial literacy on financial behavior differs significantly between urban and rural households, between men and women, and between high and low education levels. The study provides valuable insights for policy implications to enhance financial literacy, such as carrying out financial training to improve residents’ knowledge about financial aspects, which further helps to optimize household financial decision-making.

## Introduction

The last two decades have witnessed a remarkable growth in the fields of e-finance and household financial literacy. Scholars acknowledge that households with high financial literacy are more likely to become involved in stock investments (van Rooij et al., 2011; Hsiao and Tsai, 2018), have a regular bank account (Klapper et al., 2012), evaluate financial products and optimize household asset-allocation rationally (Bianchi, 2018), make reasonable pension plans (Lusardi and Mitchell, 2010; Clark et al., 2017), and become actively involved in credit card consumption and lending behavior in the current. However, limited literature has discussed the long-term and dynamic effects of financial literacy on household financial behaviors (Xu et al., 2021). Hence, our research attempts to fill this gap.

Prior studies show that media reports influence limited attention, historical information, emergency events (Barber and Odean, 2008; Aboody et al., 2009; Białowolski, 2019), and people’s ability to process information. Investors’ limited attention is related to stock price, investment behavior, financial performance, and information announcement decision (Abreu and Mendes, 2010; Blagoeva et al., 2020). Financial literacy affects the ability of individuals to process economic and financial information (Bianchi, 2018; Tian et al., 2020). Therefore, individuals with higher financial literacy actively search and discuss economic and financial information (e.g., financial reports, latest economic surveys, and even online ranking data) easily, thus affecting their financial behaviors dynamically (Choi and Choi, 2019). In fact, some financial information (e.g., M&A announcements) is relatively less accessible to the public due to information processing costs or information inertia (Illeditsch et al., 2021) relative to everyday news. Furthermore, interpreting this financial information can be tricky for individuals who lack relative financial literacy. In this scenario, does limited attention act as an underlying behavioral mechanism through which financial literacy influences financial behaviors in the long term?

We investigate the influence of household financial literacy on their limited attention and further the impact of limited attention on current, long-term, and dynamic financial behaviors (Bianchi, 2018). Then, we reveal a potential mechanism (i.e., limited attention) between financial literacy and financial behavior. We also highlight the role of limited attention as a crucial driver of financial decisions, acting as an indispensable mechanism between financial literacy and financial behavior (Aboody et al., 2009; Shaton, 2017).

Our research has several contributions. First, it contributes to the literature on the association between financial literacy and financial behavior. Even though the association between one’s financial literacy and households’ financial behaviors—especially, one’s financial market participation and allocation—has been widely studied, most of the related research focuses only on the analysis of risky assets. However, other less-risky finance behaviors, such as involvement in formal bank accounts and participation in stock markets and risk financial markets, have not been studied extensively in the existing literature. The literature mainly discusses the impact of financial literacy on current financial behaviors (Hsiao and Tsai, 2018; Tian et al., 2020), which mainly neglects the long-term and dynamic nature. Therefore, we discuss the relationship between financial literacy, limited attention, and financial behavior by focusing on the long-term and dynamic nature of the study (Xu et al., 2021).

Second, we reveal the potential mechanism (i.e., channel) of how financial literacy influences diversified financial behaviors. Previous studies mainly focus on the influencing factors of financial behavior, whereas the underlying mechanism through which financial literacy affects financial behavior remains unexplored. However, in diversified financial decisions, more types of assets should be taken into account. Individuals with higher financial literacy could actively search and process relative information (e.g., realizing economic survey data) more rationally, thus making their financial behaviors diversified and dynamic. Sufficient research revealing the mechanism through which the financial literacy affects financial behavior is lacking. Thus, this study makes a significant contribution and enhances the understanding of limited attention serving as an underlying mechanism in the association between financial literacy and households’ financial behavior. In fact, some financial information (e.g., earnings announcements) is relatively less accessible to common people due to information asymmetry and high processing costs relative to media coverage (Oliver et al., 2022).

Finally, this study conducts a heterogeneity analysis to reveal urban-rural, gender, and educational differences in the current, long-term, and dynamic effects of financial literacy on financial behavior. It provides empirical evidence for developing targeted financial literacy education. In addition, the existing literature mainly focuses on the role of macro-level policies (e.g., law and regulations) or firm information disclosure in improving investors’ limited attention (Aboody et al., 2009). However, an individual’s decisions may also be affected by their financial literacy in the both current and long-term perspective (Hsiao and Tsai, 2018). We find that limited attention can act as an underlying behavioral mechanism through which financial literacy influences financial behaviors dynamically.

The structure of this manuscript is as follows. Section 2 provides an overview of the recent literature on financial literacy and financial behaviors. Then, Section 3 describes our methodology. Next, Section 4 discusses the empirical findings. Section 5 explains the heterogeneity analysis. Finally, Section 6 concludes the study and discusses the implications.

## Literature Review

### Research on Financial Literacy and Financial Behaviors

Prior literature always considers financial literacy as a reliable and direct predictor of an individual’s economic decisions (Huston, 2010; Aren and Zengin, 2016; Struckell et al., 2022). Many studies have demonstrated that persons with higher financial literacy are more likely to participate in desirable financial behaviors, such as deposits, mutual funds, and stocks, than those with lower financial literacy (Yang et al., 2022). Additionally, people with greater financial literacy make more prudent retirement plans (Lusardi and Mitchelli, 2007; Clark et al., 2017), involvement in self-employment (Rostamkalaei et al., 2019), and sustainable investment (Habidin et al., 2020). They are also less likely to use consumer credit (Xu et al., 2020). As financial literacy has been found to be a proxy for an individual’s understanding of financial markets (Bottazzi and Lusardi, 2021), families with more financial sophistication—as assessed by wealth and education level—invest more aggressively and effectively, and they are also more likely to engage in the stock market (Abreu and Mendes, 2010; Xiao and Porto, 2017; Zou and Deng, 2019). In addition, some studies have indicated that individuals who have a higher level of financial literacy are better educated; gathering and accessing critical information more simply and affordably, they confront a lower economic threshold for financial market involvement (Xiao and Porto, 2017). On the other hand, a lack of financial literacy is associated with undesirable financial behaviors, such as low participation in financial markets (Yoong, 2010; van Rooij et al., 2012), insufficient substantial equity gains, and poor financial investment decisions (Lusardi and Mitchell, 2014). Given the connection between financial literacy and financial behavior and well-being, some research has focused on financial education programs with the overarching goal of increasing individuals’ financial literacy (Lusardi, 2019; Kaiser et al., 2021). Given the enormous variety in individuals’ financial literacy, researchers have found that financial education programs customized to specific groups are more effective than others (Lusardi and Mitchell, 2014; Cordero et al., 2020).

Even though some studies have discussed the association between one’s financial literacy and households’ financial behaviors—especially, one’s financial market participation and allocation, most of this research focuses only on the analysis of risky assets. However, other less-risky behaviors, such as involvement in formal bank accounts and participation in stock markets and risk financial markets, have not been studied extensively in academia.

### Research on Limited Attention

Attention is a scarce cognitive resource, and people pay attention to specific things at the expense of paying less attention to other things (Kahneman and Tversky, 1973). Limited attention will influence a financial anomaly when investors collect and process information to make decisions (Falkinger, 2008). Aboody et al. (2009) and Wang and Song (2022) also investigated how attention influences asset price dynamics *via* investors’ underreaction and overreaction to information.

For individual investors, time and energy constraints limit the amount of information they can obtain and analyze, thus causing difficulty for them to consider all aspects when making decisions (Falkinger, 2008). Considering the unequal consequences of losses and gains on investors’ utility levels, investors tend to overreact to risk information and overestimate risk continuity (Wang and Song, 2022). Studies have also found that individual investors rely more on the information that attracts their attention when making investment decisions and ignore the information that is useful but difficult to notice, thus leading to biased decisions (Choi and Choi, 2019). Prior studies have also shown that media coverage may influence individuals’ limited attention and reaction to specific events (Barber and Odean, 2008; Aboody et al., 2009; Białowolski, 2019) and their ability to process information. Therefore, when the news media focus on certain firms or portfolios, investors will focus on those stocks that grab their attention.

Economics is concerned with allocating a scarce resource among a variety of possibilities. The ability to pick among the options requires knowledge of the options available. The lack of information may lead to consumer ignorance with regard to possible alternatives. For example, modern consumers may receive a lot of mail with advertisements in the daytime, jostling for his or her attention. As Simon (1957) mentioned, having a lot of information turns attention into a scarce economic factor; information “consumes the attention of its recipients.” Behavior is influenced by perception regardless of the subject’s rationality. Humans have a limited capacity for processing signals, and only impulses that are sufficiently intense are perceived. In this regard, individuals tend to focus on the most prominent areas, such as the first results returned by a search engine (Sconti, 2022).

**FIGURE 1:**
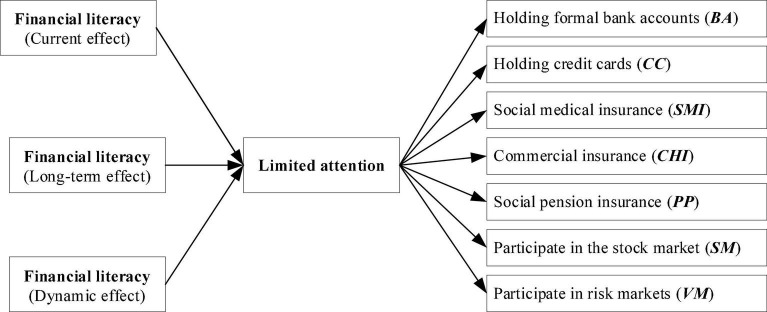
Conception framework.

However, in financial decisions, more types of assets should be taken into account. Individuals with higher financial literacy could actively search and process relative information (e.g., realizing economic survey data) more rationally, thus making their financial behaviors diversified and dynamic. We argue that limited attention serves as an underlying mechanism in the association between financial literacy and households’ financial behavior. In fact, some financial information (e.g., earnings announcements) is relatively less accessible to the public due to information asymmetry and high processing costs relative to media coverage (Oliver et al., 2022). For example, rural households with lower financial literacy may not actively participate in the financial market fairly and enjoy the benefits of financial services (Mahendru, 2020; Xu et al., 2021). In this regard, interpreting some information in daily-time financial decisions becomes challenging for individuals who lack relative financial knowledge. We also present our conception framework in Figure 1.

## Materials and Methods

### Data

We collected the data from the China Household Finance Survey (CHFS) of 2015 and 2017. The CHFS survey collects microlevel information about the household assets and liabilities, security and insurance, expenditure and income, demographic characteristics, and employment of urban and rural families. The survey samples in 2015 and 2017 collected more than 40,000 valid samples, which comprehensively portrays detailed financial situation of Chinese households. Moreover, three questions from CHFS were designed on interest rate calculation, inflation understanding, and investment risk perception, which reflect people’s financial literacy, limited attention, and financial behaviors (Zou and Deng, 2019).

### Variables

#### Financial Literacy

To reflect the financial literacy of interviewees, we used the number of questions correctly answered (Agnew and Szykman, 2005; Zou and Deng, 2019). Some studies used the factor analysis method because it reflects its financial literacy level through multidimensional index analysis (Thurstone, 1931). As such, the existing literature uses factor analysis to construct an indicator to measure respondent’s financial literacy level (Lusardi and Mitchell, 2010). Meanwhile, others used the number of questions correctly answered (Agnew and Szykman, 2005; Zou and Deng, 2019) to identify the diversified dimensions of respondents’ financial literacy. The results are relatively close when using factor analysis as well as the number of respondents’ correct answers (of three questions) to measure respondents’ financial literacy (van Rooij et al., 2011). The descriptive statistics of financial literacy in 2015 are presented in Table 1.

**TABLE 1:** Results of respondents’ answering of financial questions.

Frequency	0	1	2	3	Sum
Correctly	11.67%	46.01%	31.55%	10.77%	1.414
Incorrectly	40.09%	37.24%	21.07%	1.16%	0.842
Unknown	57.26%	17.60%	18.63%	6.50%	0.744

*Calculated by the data of Chinese Household Financial Survey in 2015. 0, 1, 2, 3 refer to respondents’ answer question correctly (incorrectly/unknown) one time, two times, three times.*

Table 1 shows that only 10.77% of the households answered all three questions (interest rate calculation, inflation, and venture market) correctly. The mean value of the number of questions they answered correctly was just 1.414, which was relatively lower than that of the residents from developed countries. Nearly 6.50% of the households knew nothing about these three questions. This outcome shows that Chinese households are seriously short of financial literacy and have not yet understood some basic financial information. Hence, financial literacy needs to be further strengthened in the future.

#### Limited Attention

We reviewed the respondents’ attention to economic and financial information in the CHFS questionnaire (Zou and Deng, 2019). *Limited attention* was a dummy variable assigned to 1 if respondents were very concerned about economic and financial information, otherwise 0 to general, less concerned, and never concerned. If respondents possessed a certain level of financial literacy, they would pay more attention to financial information and understand the financial market and financial products. Resultantly, their financial decisions would be more rational and optimized.

#### Financial Behaviors

We considered two aspects of financial behaviors: acceptance of financial products and participation in the financial market. In terms of acceptance of financial products, we examined it by inquiring whether they held formal bank accounts and credit cards and whether they participated in social, medical insurance, commercial insurance, and pension plans (Stolper and Walter, 2017; Bianchi, 2018; Song, 2020). Meanwhile, we investigated financial market participation by reviewing whether households had been involved in the stock market and venture market (=1); otherwise, it would be 0 (Hsiao and Tsai, 2018; Song, 2020). The details of financial behavior are presented in Table 2.

**TABLE 2:** Definition about financial behavior.

Types of financial behavior		Questionnaire	Description
Residents’ acceptance of financial products	Holding formal bank accounts	Currently, does your family have an RMB current deposit account?	It is a dummy variable that takes 1 if the respondent answered that he holds an RMB current deposit account or holds an unexpired RMB time deposit; otherwise, 0.
		Currently, does your family have any outstanding RMB time deposits?	
	Holding credit cards	Does your family use credit cards? Inactive credit cards are not included.	It is a dummy variable that takes 1 if the respondent answers yes; otherwise, 0.
	Social medical insurance	Do you currently have social medical insurance, excluding major illness planning and commercial medical insurance?	It is a dummy variable that takes 1 if the respondent has social medical insurance; otherwise, 0.
	Commercial insurance	Have you participated in commercial insurance?	It is a dummy variable that takes 0 if the respondent answers none, which means no commercial insurance; otherwise, 0.
	Pension plans	Which of the following social pension insurance do you receive after retirement/retirement?	It is a dummy variable that takes 0 if the respondent answers none, which means no social pension insurance; otherwise, 0.
Residents’ participation in the financial market	Involvement in stock market	Currently, does your family hold a stock account?	It is a dummy variable that takes 1 if the respondent answers yes; otherwise, 0.
	Involvement in venture market	Currently, does your family hold stocks/funds/bonds/financial derivatives/bank wealth management products/non-RMB assets/gold accounts?	It is a dummy variable that takes 1 if the respondent answers that he or she has one of the financial accounts; the value of participating in the risky financial market is assigned 1; otherwise, 0.

*This table reports the financial behavior of the main variables used in our multivariate analysis for our sample of households on the China Household Financial Survey Data in 2015.*

#### Control Variables

This study considered various control variables based on the prior studies (Korkmaz et al., 2021; Xu et al., 2021), including age, age square, education level, marital status (*Married*), health status (*Health*), risk attitude, and other demographic characteristics (Lei et al., 2013; Zhao et al., 2016; Shu, 2018; Tang et al., 2021; Zhao and Qu, 2021). Family characteristic variables included the following: *Number of children in the family* (age<15), *number of elderly in the family* (age > 60), *family size, homeownership, owning two or more houses, total family income* (natural logarithm), and *total family assets* (take natural logarithm). *Homeownership* was a dummy variable assigned a value of 1 if they had their own house, otherwise 0. The variable definitions are reported in Supplementary Table 1. Provincial dummy variables were also included to control the regional difference. The sample with missing value was eliminated, and we also winsorized our sample at 0.5% for *total family assets* and *total family income*. The descriptive statistics of the 2015 survey are presented in Supplementary Table 1.

### Estimation Model

First, the nature of our dependent variable, financial behavior being a discrete binary variable, suggests using a *Probit* model to examine the relationship between financial literacy and financial behavior (as Equation 1).


(1) $$Prob\left(Y_{i}=1|X_{i}\right)=\phi \left(\alpha +\beta_{1}X_{i\,1}+\beta_{2}X_{i\,2}+\mu \right),$$

where *Y_i* represents the financial literacy of residents in 2015, *X*_*i1*_ is the total score of residents’ financial literacy in 2015, and *X*_*i2*_ is the control variable in 2015. To be specific, when studying the current impact of financial literacy on behavior, financial literacy refers to the household financial literacy level in 2015, whereas the dependent variable is the financial behavior of households in 2015. All of our regression results cluster robust standard errors to the community level in this study.

In studying the long-term impact of financial literacy on financial behavior, *Financial Literacy* refers to the household financial literacy level in 2015, whereas the dependent variable was the financial behavior of households in 2017.

We employed the *OrderedProbit* model to test the dynamic impact of financial literacy on financial literacy as captured by Equation 3. *Y_i* represented the change in financial behavior (Xu et al., 2021). To be specific, *Y_i* was equal to 1 if residents engaged in financial behavior in 2017 but did not do so in 2015 (i.e., financial behavior = 0 in 2015 but financial behavior = 1 in 2017), *Y_i* was equal to 0 if the residents’ financial behavior had not changed (i.e., financial behavior = 1 or 0 both in 2015 and 2017), *Y*_*i*_ was equal to −1 if residents did not engage in financial behavior in 2017 but did so in 2015 (i.e., financial behavior = 1 in 2015 but financial behavior = 0 in 2017). *X*_*i1*_ (financial literacy) denoted the total score of residents’ financial literacy in 2015, and *X*_*i1*_ represented the control variable in 2015. *u_i* was a random error term, which was assumed to follow a standard normal distribution. The form of *F*(⋅) function is presented as follows:


(2) $$Y_{i}=F\,\left(a+\beta \,X_{i\,1}+\phi_{i}\,X_{i\,2}+u_{i}\right),$$

(3) $$F\,\left(Y_{i}^{*}\right)=\left\{ \begin{array}{cc} -1\; & Y_{i}^{*}<\mu_{1}\\ 0\; & \mu_{1}<Y_{i}^{*}<\mu_{2}\\ \vdots \; & \vdots \\ r\; & Y_{i}^{*}>\mu_{r-1}, \end{array}\right.$$

where y* is the latent variable of *Y* and μ_1_ < μ_2_ < ⋯ < μ_*r*−1_ is the tangent point. y* satisfies the following:


(4) $$Y_{i}^{*}=\beta \,X_{i\,1}+\phi_{i}\,X_{i\,1}+u_{i}.$$

## Results

### Descriptive Analysis

Supplementary Table 1 shows that 81.91 and 27.09% of the individuals held formal bank accounts and credit cards in our sample, respectively. Supplementary Table 1 illustrates that 16.36, 19.70, 92.35, 11.83, and 80.10% of individuals participated in the stock market, risk financial market, social, medical insurance, commercial insurance, and social pension insurance, respectively. The level of household credit card holding, stock market participation, and commercial insurance participation was relatively low, and a significant gap remained in the level of financial literacy among different families.

### The Relationship Between Financial Literacy and Financial Behavior

Table 3 reports the regression results of the current, long-term, and dynamic effects of residents’ financial literacy on financial behaviors (i.e., direct effect) (Xu et al., 2021). In panel A, we found that residents’ financial literacy has a positive association with these financial behaviors (e.g., owning commercial bank accounts, participation in stock markets and risk investment, social medical insurance, commercial insurance, pension plans, and holding credit cards) in the current. In panel B, we found that residents’ social medical insurance was positive with involvement in these financial behaviors except social medical insurance in the long term. In panel C, we found that financial literacy is helpful to improve the residents’ involvement in some financial products except social medical insurance. This result shows that financial literacy has current, long-term, and dynamic effects on financial behavior.

**TABLE 3:** The impact of financial literacy on financial behavior.

Variable	(1)	(2)	(3)	(4)	(5)	(6)	(7)
	
	BA	SM	VM	SMI	CHI	PP	CC
**Panel A: Current effect**							
FL	0.0250*** (7.3033)	0.0214*** (7.3730)	0.0260*** (8.6132)	0.0042* (1.7044)	0.0104*** (3.8582)	0.0084** (2.3510)	0.0224*** (6.5417)
GENDER	0.0086 (1.3946)	−0.0160*** (−3.1202)	−0.0228*** (−4.1154)	−0.0022 (−0.5094)	−0.0276*** (−5.8284)	−0.0439*** (−6.7138)	−0.0283*** (−4.5511)
AGE	−0.0030** (−2.4311)	0.0098*** (8.7111)	0.0096*** (8.3553)	0.0052*** (5.8506)	0.0163*** (11.1588)	0.0177*** (14.0363)	0.0040** (2.5622)
AGE2	0.0000** (2.2963)	−0.0001*** (−8.5055)	−0.0001*** (−8.1827)	−0.0000*** (−2.9159)	−0.0002*** (−11.9971)	−0.0001*** (−8.4202)	−0.0001*** (−4.7243)
EDU	0.0192*** (9.9787)	0.0345*** (22.8244)	0.0402*** (25.7364)	0.0081*** (5.7152)	0.0099*** (7.0389)	0.0471*** (22.0239)	0.0434*** (25.6604)
MAR	−0.0213*** (−2.6034)	0.0126* (1.6674)	0.0078 (0.9684)	0.0019 (0.3020)	−0.0244*** (−3.2936)	0.0235*** (2.8753)	0.0122 (1.3159)
HEALTH	−0.0018 (−0.3437)	−0.0092** (−2.0678)	−0.0127*** (−2.6566)	−0.0125*** (−3.1939)	0.0004 (0.0979)	−0.0043 (−0.7679)	0.0041 (0.7353)
FL	0.0033 (0.4650)	−0.0453*** (−6.8382)	−0.0482*** (−6.9302)	0.0256*** (5.0769)	−0.0083 (−1.3247)	0.0397*** (5.2141)	0.0117 (1.5084)
OLD	0.0058 (1.4544)	−0.0047 (−1.3146)	−0.0029 (−0.7661)	−0.0007 (−0.2537)	−0.0020 (−0.5976)	0.0103** (2.4938)	−0.0158*** (−3.8018)
CHILD	−0.0101*** (−2.8194)	−0.0128*** (−3.5205)	−0.0176*** (−4.4348)	0.0036 (1.2371)	−0.0085** (−2.5730)	−0.0140*** (−3.6116)	0.0169*** (4.1105)
FS	−0.0048** (−2.1596)	0.0015 (0.7427)	0.0017 (0.8060)	−0.0053*** (−3.6538)	−0.0015 (−0.8696)	−0.0073*** (−2.9888)	0.0027 (1.1900)
RL	−0.0102 (−1.0844)	0.0937*** (14.4795)	0.0914*** (13.2775)	−0.0065 (−1.0817)	0.0070 (1.0747)	0.0072 (0.8437)	0.0262*** (3.1191)
RA	−0.0032 (−0.5100)	−0.0505*** (−9.5069)	−0.0551*** (−9.8938)	0.0009 (0.2326)	−0.0142*** (−2.8322)	0.0094 (1.5173)	−0.0611*** (−9.7532)
HO	0.0570*** (5.1118)	0.0250*** (3.0016)	0.0249*** (2.6725)	−0.0261*** (−4.0272)	−0.0034 (−0.4144)	−0.0341*** (−3.4652)	0.0283*** (2.7379)
OH	−0.1522*** (−13.2082)	−0.1247*** (−11.7437)	−0.1416*** (−12.7305)	0.0252*** (3.4769)	−0.0431*** (−4.6807)	0.0087 (0.7784)	−0.1413*** (−12.0164)
TA	0.0572*** (24.7530)	0.0639*** (24.5514)	0.0746*** (27.4203)	0.0026 (1.5428)	0.0390*** (17.3675)	0.0158*** (6.3655)	0.0754*** (28.1949)
TI	0.0152*** (14.0838)	0.0125*** (7.9373)	0.0148*** (8.9239)	0.0061*** (8.5078)	0.0065*** (5.0091)	0.0181*** (16.1182)	0.0152*** (8.9275)
Province dummy	Yes	Yes	Yes	Yes	Yes	Yes	Yes
*N*	20,577	20,577	20,577	20,577	20,577	20,577	20,577
Pseudo *R*^2^	0.1282	0.2687	0.2703	0.0526	0.1098	0.1303	0.2435
**Panel B: Long**−**term effect**							
FL	0.0056** (2.0433)	0.0177*** (5.2473)	0.0233*** (6.7959)	0.0010 (0.3872)	0.0180*** (5.8490)	0.0422*** (12.7653)	0.0171*** (4.1683)
GENDER	−0.0002 (−0.0484)	−0.0180*** (−3.0725)	−0.0256*** (−4.2358)	−0.0007 (−0.1365)	−0.0191*** (−3.4971)	−0.0222*** (−3.3209)	−0.0199** (−2.4751)
AGE	−0.0009 (−0.8451)	0.0052*** (3.6470)	0.0050*** (3.3090)	0.0041*** (4.3828)	0.0075*** (5.0889)	0.0081*** (5.7208)	−0.0006 (−0.2829)
AGE2	0.0000 (0.1846)	−0.0001*** (−3.5816)	−0.0000*** (−3.1885)	−0.0000*** (−3.4083)	−0.0001*** (−5.6717)	−0.0000** (−2.2699)	−0.0000 (−1.5872)
EDU	0.0133*** (7.7188)	0.0288*** (17.8818)	0.0341*** (19.7065)	0.0074*** (5.0488)	0.0120*** (7.2802)	0.0402*** (17.5464)	0.0438*** (20.0228)
MAR	0.0090 (1.4192)	0.0052 (0.6070)	0.0033 (0.3680)	0.0123* (1.8813)	−0.0079 (−0.9476)	0.0138 (1.5362)	−0.0005 (−0.0423)
HEALTH	0.0026 (0.6298)	0.0030 (0.6015)	0.0044 (0.8237)	−0.0073* (−1.7726)	−0.0038 (−0.7845)	0.0009 (0.1627)	0.0117* (1.7140)
FL	0.0030 (0.6060)	−0.0393*** (−5.2439)	−0.0450*** (−5.7142)	0.0033 (0.6096)	0.0139* (1.8983)	0.0005 (0.0702)	0.0220** (2.3555)
OLD	−0.0009 (−0.3016)	−0.0094** (−2.5532)	−0.0086** (−2.1812)	0.0007 (0.2464)	−0.0000 (−0.0016)	0.0285*** (6.7746)	−0.0260*** (−5.1502)
CHILD	0.0064** (2.2638)	−0.0165*** (−4.1006)	−0.0179*** (−4.1903)	−0.0045 (−1.6197)	0.0052 (1.5235)	−0.0127*** (−3.4850)	0.0128*** (2.5879)
FS	−0.0016 (−1.0925)	0.0006 (0.2753)	0.0001 (0.0646)	0.0023 (1.4738)	0.0007 (0.3487)	−0.0054** (−2.2875)	0.0048* (1.7562)
RL	0.0092 (1.1594)	0.0621*** (8.1442)	0.0653*** (8.0663)	−0.0108 (−1.5490)	0.0181** (2.4044)	0.0019 (0.2121)	0.0213* (1.8723)
RA	−0.0025 (−0.5352)	−0.0358*** (−6.3062)	−0.0341*** (−5.8246)	0.0024 (0.4967)	−0.0101* (−1.7574)	0.0116* (1.8571)	−0.0467*** (−6.1550)
HO	0.0025 (0.2516)	0.0018 (0.1728)	0.0125 (1.0870)	−0.0172** (−2.0232)	0.0311*** (3.1985)	−0.0222** (−2.2274)	0.0492*** (3.4668)
OH	−0.0139 (−1.5990)	−0.0882*** (−7.0959)	−0.1036*** (−7.8899)	0.0140 (1.6187)	−0.0254** (−2.2522)	0.0006 (0.0492)	−0.1119*** (−7.3804)
TA	0.0100*** (5.8611)	0.0453*** (15.0108)	0.0545*** (17.4893)	0.0030 (1.6280)	0.0230*** (9.2693)	0.0068*** (2.7537)	0.0602*** (18.0693)
TI	0.0056*** (7.1597)	0.0086*** (4.6869)	0.0087*** (4.5535)	0.0014 (1.5155)	0.0015 (1.1092)	0.0050*** (4.3685)	0.0122*** (5.8347)
Province dummy	13,869	13,869	13,869	13,869	13,799	14,782	13,869
*N*	Yes	Yes	Yes	Yes	Yes	Yes	Yes
Pseudo *R*^2^	0.0785	0.2265	0.2343	0.0225	0.0843	0.1305	0.1942
**Panel C: Dynamic effect**							
FL	0.0573*** (3.7732)	0.0019** (2.0441)	0.0020** (2.1075)	−0.0132 (−0.7394)	0.0303* (1.8719)	0.1181*** (7.9442)	0.0038** (2.2508)
GENDER	−0.0085 (−0.2988)	−0.0008 (−0.0214)	0.0142 (0.4174)	−0.0135 (−0.3988)	0.0488 (1.5103)	0.0510* (1.8880)	0.0476 (1.6195)
AGE	0.0098* (1.6628)	−0.0031 (−0.4322)	−0.0011 (−0.1624)	−0.0135* (−1.8579)	−0.0168*** (−2.9020)	−0.0337*** (−5.8797)	−0.0057 (−1.0292)
AGE2	−0.0001** (−1.9953)	0.0000 (0.4614)	0.0000 (0.1289)	0.0001 (0.9775)	0.0002*** (3.8282)	0.0003*** (4.9660)	0.0000 (0.7583)
EDU	−0.0236*** (−3.0091)	−0.0263** (−2.3593)	−0.0334*** (−3.2841)	−0.0059 (−0.6035)	0.0067 (0.7203)	−0.0197** (−2.5018)	0.0026 (0.2985)
MAR	0.0663 (1.6088)	−0.0398 (−0.8307)	−0.0317 (−0.7146)	0.0654 (1.2757)	0.0619 (1.4983)	−0.0592 (−1.5631)	−0.0344 (−0.8766)
HEALTH	−0.0073 (−0.3023)	0.0678** (2.5061)	0.0847*** (3.3260)	0.0075 (0.2733)	−0.0329 (−1.2259)	−0.0032 (−0.1371)	0.0432* (1.8762)
FL	−0.0061 (−0.1837)	0.0120 (0.3363)	−0.0151 (−0.4464)	−0.1011*** (−2.7904)	0.0457 (1.3891)	−0.0916*** (−2.8858)	0.0090 (0.3072)
OLD	−0.0284 (−1.6444)	−0.0156 (−0.7814)	−0.0085 (−0.4645)	0.0019 (0.0988)	0.0194 (1.1068)	0.0571*** (3.4691)	−0.0293* (−1.7631)
CHILD	0.0549*** (3.3485)	−0.0020 (−0.1269)	0.0059 (0.3801)	−0.0324 (−1.6059)	0.0649*** (3.7218)	0.0069 (0.3879)	−0.0056 (−0.3604)
FS	0.0021 (0.2307)	−0.0053 (−0.5362)	−0.0046 (−0.4944)	0.0331*** (3.2954)	0.0079 (0.8480)	0.0057 (0.6306)	0.0057 (0.6153)
RL	0.0307 (0.8067)	−0.2557*** (−4.4462)	−0.1735*** (−3.3584)	0.0260 (0.5111)	0.0532 (1.0721)	−0.0068 (−0.1769)	−0.0468 (−1.0644)
RA	−0.0115 (−0.3998)	0.0455 (1.3826)	0.0548* (1.7566)	0.0222 (0.6975)	0.0307 (0.9938)	−0.0066 (−0.2386)	0.0154 (0.5517)
HO	−0.1913*** (−3.8732)	−0.0893 (−1.4845)	−0.0211 (−0.3567)	0.0474 (0.7088)	0.1347** (2.1533)	−0.0493 (−0.9359)	−0.0126 (−0.2373)
OH	0.4748*** (9.1763)	0.1756*** (2.9467)	0.1436** (2.3733)	−0.0616 (−0.8942)	0.0958* (1.9356)	−0.0366 (−0.6783)	0.0403 (0.8896)
TA	−0.1662*** (−14.9147)	−0.0787*** (−6.7650)	−0.0674*** (−6.0683)	0.0050 (0.3822)	−0.0514*** (−4.9855)	−0.0267** (−2.5636)	−0.0317*** (−3.4332)
TI	−0.0336*** (−5.2540)	−0.0180*** (−2.6936)	−0.0208*** (−3.2864)	−0.0217*** (−2.8665)	−0.0159*** (−2.6871)	−0.0557*** (−9.3590)	−0.0042 (−0.8159)
Province dummy	Yes	Yes	Yes	Yes	Yes	Yes	Yes
*N*	13,869	13,869	13,869	13,869	13,799	13,799	13,869
Pseudo *R*^2^	0.0606	0.0168	0.0158	0.0049	0.0087	0.0241	0.0045

This table presents regression results for the impact of financial literacy on financial behavior. The dependent variable is shown above each column. Standard errors are single-clustered by community level and Z-statistics, which are shown in parentheses. Variable definitions are presented in Supplementary Table 1. *, **, and *** indicate significance at the 10, 5, and 1% levels, respectively.

In addition, endogenous issues may arise due to omitted variables and reverse causality (Hill et al., 2020; Yang et al., 2020). The financial literacy of residents may be affected by participation in financial markets (Tang et al., 2021; Zhao and Qu, 2021). Their understanding of financial products will be deepened through continuous learning and participation in the stock markets. Based on prior studies (Kim and Lee, 2017; Zhao and Qu, 2021), we selected the average financial literacy level of people living in the same village (community) aside from themselves as the instrumental variable (IV) of respondents’ financial literacy, whereas the IV-2SLS method was used to estimate our models (Semadeni et al., 2014; Hill et al., 2020). Residents could learn and accumulate specific financial literacy by interacting with other people (Zhao and Qu, 2021). The average financial literacy of villages (communities) was exogenous relative to the interviewees’ participation in financial behaviors. Therefore, the average financial literacy of other people in the same village (community) was the instrumental variable (IV) of respondents’ financial literacy. The results of IV-2SLS are presented in Table 4.

**TABLE 4:** IV estimation of financial literacy and financial behavior.

Variable	(1)	(2)	(3)	(4)	(5)	(6)	(7)
	
	BA	SM	VM	SMI	CHI	PP	CC
**Panel A: Current effect**							
FL	0.0835*** (2.7471)	0.3576*** (8.4561)	0.3629*** (9.0569)	0.0969** (2.4776)	0.1650*** (4.1833)	0.0439** (2.4563)	0.2121*** (6.1869)
Controls	Yes	Yes	Yes	Yes	Yes	Yes	Yes
Province dummy	Yes	Yes	Yes	Yes	Yes	Yes	Yes
*N*	20,577	20,577	20,577	20,577	20,577	20,577	20,577
***IVorderProbit* Stage 1:**							
*F* values of IV	420.23***						
*T* values of IV	68.89***						
DWH value (*p*-value)	10.88*** (0.0006)	37.24*** (0.0000)	39.95*** (0.0000)	13.27*** (0.0003)	8.69*** (0.0032)	10.12*** (0.0008)	15.10*** (0.0001)
**Panel B: Long-term effect**							
FL	0.0882* (1.9035)	0.3735*** (7.0527)	0.3971*** (7.8652)	−0.0740 (−1.5294)	0.1575*** (3.3028)	0.1361*** (3.7665)	0.1635*** (4.3066)
Controls	Yes	Yes	Yes	Yes	Yes	Yes	Yes
Province dummy	Yes	Yes	Yes	Yes	Yes	Yes	Yes
*N*	13,869	13,869	13,869	13,869	13,869	13,869	13,869
***IVorderProbit* Stage 1:**							
*F* values of IV	297.97***						
*T* values of IV	59.85***						
DWH value (*p*-value)	10.88*** (0.0015)	28.47*** (0.0000)	32.26*** (0.0000)	3.71* (0.0541)	10.85*** (0.0016)	9.10*** (0.0025)	8.22*** (0.0041)
**Panel C: Dynamic effect**							
FL	0.0462** (2.2620)	0.2069*** (3.6882)	0.2481*** (4.7625)	0.0452 (1.0143)	0.1002** (2.1393)	0.0630* (1.7506)	0.0282* (1.7101)
Controls	Yes	Yes	Yes	Yes	Yes	Yes	Yes
Province dummy	Yes	Yes	Yes	Yes	Yes	Yes	Yes
*N*	13,869	13,869	13,869	13,869	13,869	13,869	13,869
***IVorderProbit* Stage 1:**							
*F* values of IV	330.73***						
*T* values of IV	57.18***						
DWH value (*p*-value)	10.51*** (0.0016)	7.31*** (0.0069)	12.25*** (0.0005)	5.81** (0.0159)	18.32*** (0.0000)	3.16* (0.0757)	12.34*** (0.0004)

This table presents regression results for IV estimation of financial literacy on financial behavior. The dependent variable is shown above each column. Controls include GENDER, AGE, AGE2, EDU, MAR, HEALTH, FL, OLD, CHILD, FS, RL, RA, HO, OH, TA, TI. The DWH exogenous test refers to the Durbin–Wu–Hausman test, and it is mainly to test whether there is endogeneity between financial literacy and financial behaviors. The first-stage estimation of the F value and instrumental variable T value is mainly to test whether the instrumental variables we select are weak. Standard errors are single-clustered by community level and Z-statistics, which are shown in parentheses. Variable definitions are presented in Supplementary Table 1. *, **, and *** indicate significance at the 10, 5, and 1% levels, respectively.

The results of the one-stage estimated *F* value and the instrumental variable *t* value in Table 4 show that no weak instrumental variable problem was detected, and the instrumental variables we selected were appropriate. The second-stage regression results of Table 4 revealed that the coefficients of financial literacy are positive and all pass the statistical significance test. Endogenous test results show that financial literacy has a causal relationship with current, long-term, and dynamic financial behavior. The results of Panel A show that after controlling for other factors, financial literacy has a significant positive impact on financial behaviors, such as having formal bank accounts, stock market participation, risk market participation, medical insurance participation, commercial insurance participation, pension plans, and holding credit cards in the current. Panel B and Panel C list the long-term and dynamic impact estimation results in detail. We found that financial literacy helps to improve the residents’ involvement in some financial products, except commercial insurance in long-term and dynamic impacts.

### The Impact of Financial Literacy on Residents’ Limited Attention

Table 5 reports the regression results of the current, long-term, and dynamic effects of financial literacy on residents’ limited attention (Xu et al., 2021). In Model 1, the marginal effect of *FL* (*b* = 0.0704) was positive and significant at the 1% level (Wiersema and Bowen, 2009), thus indicating that the higher the financial literacy of residents is, the more likely they are concerned about economic and financial information (i.e., *LA* = 1) in the current. In Model 2, the marginal effect of *FL* (*b* = 0.0383) was positive and significant at the 1% level, thus indicating that the higher the level of financial literacy is, the more likely they are concerned about economic and financial information (i.e., *LA* = 1) in the long term. In Model 3, the marginal effect of *FL* (*b* = 0.0453) was positive and significant at the 1% level, thus indicating that financial literacy is helpful to improve the residents’ limited attention (i.e., dynamic effect). Residents with higher levels of financial literacy may do well in searching, analyzing, and processing financial and economic information and gradually enhance their limited attention.

**TABLE 5:** The impact of financial literacy on limited attention.

Variable	(1)	(2)	(3)	(4)	(5)	(6)
	
	LA Current	LA Long-term	LA Dynamic	LA Current IV	LA Long-term IV	LA Dynamic IV
FL	0.0704*** (16.8057)	0.0383*** (7.6395)	0.0453*** (3.5693)	0.3137*** (11.2583)	0.1691*** (5.3502)	0.0948*** (2.9056)
Controls	Yes	Yes	Yes	Yes	Yes	Yes
Province dummy	Yes	Yes	Yes	Yes	Yes	Yes
*N*	20,577	13,869	13,869	20,577	13,869	13,869
Pseudo *R*^2^	0.1068	0.0751	0.0066			
*IVorderProbit* Stage 1:						
*F* values of IV				420.23***	297.97***	330.73***
*T* values of IV				68.89***	59.85***	57.18***
DWH value (*p*-value)				19.69*** (0.0000)	4.27** (0.0388)	1.44 (0.2296)

This table presents regression results for the impact of financial literacy on limited attention. The dependent variable is shown above each column. Controls include GENDER, AGE, AGE2, EDU, MAR, HEALTH, FL, OLD, CHILD, FS, RL, RA, HO, OH, TA, TI. Columns (4)-(6) are listed as IV estimation results. The DWH exogenous test refers to the Durbin–Wu–Hausman test, and it is mainly to test whether there is endogeneity between financial literacy and financial behaviors. The first-stage estimation of the F value and instrumental variable T value is mainly to test whether the instrumental variables we select are weak. Standard errors are single-clustered by community level and Z-statistics, which are shown in parentheses. Variable definitions are presented in Supplementary Table 1. *, **, and *** indicate significance at the 10, 5, and 1% levels, respectively.

In addition, financial literacy and limited attention may have reverse causality and lead to endogeneity problems. Residents’ financial literacy may be affected by their usual attention to economic and financial-related information (Tang et al., 2021). Nevertheless, we opted for the average level of financial literacy of people living in the same village (community) other than the participants as the instrumental variable (IV) of the respondents’ financial literacy and the IV-2SLS method to estimate our model (Semadeni et al., 2014; Hill et al., 2020). The results of IV-2SLS are listed in columns (4–6) in Table 5. We can find that after dealing with endogenous problems, the current, long-term, and dynamic effects of financial literacy on limited attention are still positive and significant, which indicates that no reverse causality was observed between the financial literacy and limited attention of residents.

### The Impact of Residents’ Limited Attention on Financial Behavior

Table 6 reports the regression results of the current, long-term, and dynamic effects of residents’ limited attention on financial behaviors (Xu et al., 2021). In panel A, *LA* refers to respondents’ attention to economic and financial information in 2015, whereas financial behaviors (including *BA, SM, VM, SMI, CHI, PP*, and *CC*) refer to their involvement in some financial products in 2015. We found that residents’ limited attention has a positive association with these financial behaviors (e.g., owning commercial bank accounts, participation in stock markets and risk investment, commercial insurance, pension plans, and holding credit cards) except social medical insurance in the current. As we know, social medical insurance is a type of compulsory service in most cities. Thus, the relationship between limited attention and social medical insurance is not significant. In panel B, *LA* refers to respondents’ attention to economic and financial information in 2015, whereas *Financial behavior* refers to their involvement in some financial products in 2017. We found that residents’ limited attention has a positive association with these financial behaviors except social medical insurance in the long term. In panel C, *LA* refers to changes in respondents’ concerns about economic and financial information between 2015 and 2017. We found that limited attention helps to increase residents’ acceptance of financial products and participation in financial markets. This result shows that the limited attention of residents can significantly and positively improve the decision-making of residents’ financial behavior. Residents pay much attention to economic and financial information. Hence, they can learn more about financial products and policy dynamics to make more appropriate and reasonable financial behavior decisions.

**TABLE 6:** The impact of limited attention on financial behavior.

Variable	(1)	(2)	(3)	(4)	(5)	(6)	(7)
	
	BA	SM	VM	SMI	CHI	PP	CC
**Panel A: Current effect**							
LA	0.0291*** (5.2477)	0.0972*** (20.8206)	0.1043*** (21.6129)	0.0044 (1.0693)	0.0253*** (5.5241)	0.0221*** (3.8774)	0.0502*** (8.8078)
Controls	Yes	Yes	Yes	Yes	Yes	Yes	Yes
Province dummy	Yes	Yes	Yes	Yes	Yes	Yes	Yes
*N*	20,577	20,577	20,577	20,577	20,577	20,577	20,577
Pseudo *R*^2^	0.1266	0.2901	0.2893	0.0524	0.1108	0.1307	0.2450
**Panel B: Long-term effect**							
LA	0.0122*** (2.7927)	0.0666*** (13.0272)	0.0699*** (13.1112)	−0.0006 (−0.1505)	0.0203*** (4.0928)	0.0183*** (3.2222)	0.0431*** (6.1195)
Controls	Yes	Yes	Yes	Yes	Yes	Yes	Yes
Province dummy	Yes	Yes	Yes	Yes	Yes	Yes	Yes
*N*	13,869	13,869	13,869	13,869	13,869	13,869	13,869
Pseudo *R*^2^	0.0790	0.2396	0.2442	0.0225	0.0820	0.1171	0.1955
**Panel C: Dynamic effect**							
LA	0.0592** (2.3682)	0.1345*** (4.3333)	0.1302*** (4.3796)	0.0519* (1.7470)	0.0122** (2.4586)	0.0374** (2.5366)	0.0202* (1.8058)
Controls	Yes	Yes	Yes	Yes	Yes	Yes	Yes
Province dummy	Yes	Yes	Yes	Yes	Yes	Yes	Yes
*N*	13,869	13,869	13,869	13,869	13,869	13,869	13,869
Pseudo *R*^2^	0.0464	0.0234	0.0188	0.0063	0.0092	0.0164	0.0140

This table presents regression results for the impact of limited attention on financial behavior. The dependent variable is shown above each column. Controls include GENDER, AGE, AGE2, EDU, MAR, HEALTH, FL, OLD, CHILD, FS, RL, RA, HO, OH, TA, TI. Standard errors are single-clustered by community level and Z-statistics, which are shown in parentheses. Variable definitions are presented in Supplementary Table 1. *, **, and *** indicate significance at the 10, 5, and 1% levels, respectively.

Similarly, an endogenous relationship may also exist between residents’ limited attention and financial behavior. On the one hand, as residents pay increased attention to economic and financial-related information, their understanding of financial products, financial services, and financial markets will become clearer, thus facilitating them to make corresponding financial behavior decisions. On the other hand, given the increase in financial behavior decision-making, residents may select economic and financial information that is more related to their financial behavior to improve their financial behavior further. We selected the average limited attention level of people living outside the same village (community) as an instrumental variable (IV) for the respondents’ limited attention and employed the IV-2SLS method to estimate our model (Semadeni et al., 2014; Hill et al., 2020). The results of IV-2SLS are listed in Table 7. After dealing with endogenous problems, the current, long-term, and dynamic effects of residents’ limited attention on financial behavior are still positive and significant. This result is consistent with the estimates in Table 6, with only a small change in the marginal effect coefficient of limited attention. The results in Table 7 show that the limited attention of residents still has a significant impact on financial behavior after controlling for endogenous problems.

**TABLE 7:** IV estimation of limited attention on financial behavior.

Variable	(1)	(2)	(3)	(4)	(5)	(6)	(7)
	
	BA	SM	VM	SMI	CHI	PP	CC
**Panel A: Current effect**							
LA	0.1251* (1.8348)	0.9648*** (11.1101)	0.9252*** (11.3280)	0.1584* (1.8460)	0.2915*** (3.5956)	0.3267*** (4.8110)	0.6681*** (9.3330)
Controls	Yes	Yes	Yes	Yes	Yes	Yes	Yes
Province dummy	Yes	Yes	Yes	Yes	Yes	Yes	Yes
*N*	20,577	20,577	20,577	20,577	20,577	20,577	20,577
***IVorderProbit* Stage 1:**							
*F* values of IV	308.43***						
*T* values of IV	50.43***						
DWH value (*p*-value)	10.00*** (0.0003)	24.80*** (0.0000)	25.91*** (0.0000)	5.59** (0.0180)	3.77* (0.0522)	13.67*** (0.0002)	48.10*** (0.0000)
**Panel B: Long-term effect**							
LA	0.4621*** (4.7217)	1.1338*** (12.9054)	1.1298*** (13.5538)	0.1064 (1.1465)	0.4618*** (5.5595)	0.4434*** (5.8777)	0.6497*** (9.6309)
Controls	Yes	Yes	Yes	Yes	Yes	Yes	Yes
Province dummy	Yes	Yes	Yes	Yes	Yes	Yes	Yes
*N*	13,869	13,869	13,869	13,869	13,869	13,869	13,869
***IVorderProbit* Stage 1:**							
*F* values of IV	232.45***						
*T* values of IV	51.91***						
DWH value (*p*-value)	10.84*** (0.0062)	34.49*** (0.0000)	40.90*** (0.0000)	10.05*** (0.0068)	7.11*** (0.0077)	18.42*** (0.0000)	22.56*** (0.0000)
**Panel C: Dynamic effect**							
LA	0.0110** (2.1391)	0.5661*** (5.0823)	0.6098*** (5.9330)	0.0019** (2.0196)	0.2306** (2.4510)	0.2711*** (3.3416)	0.0436** (2.5270)
Controls	Yes	Yes	Yes	Yes	Yes	Yes	Yes
Province dummy	Yes	Yes	Yes	Yes	Yes	Yes	Yes
*N*	13,869	13,869	13,869	13,869	13,869	13,869	13,869
***IVorderProbit* Stage 1:**							
*F* values of IV	202.49***						
*T* values of IV	42.28***						
DWH value (*p*-value)	1.83 (0.1757)	6.25** (0.0124)	14.63*** (0.0001)	24.25*** (0.0000)	23.06*** (0.0000)	6.54** (0.0106)	20.86*** (0.0000)

This table presents regression results for the IV estimation of limited attention on financial behavior. The dependent variable is shown above each column. Controls include GENDER, AGE, AGE2, EDU, MAR, HEALTH, FL, OLD, CHILD, FS, RL, RA, HO, OH, TA, TI. Columns (4–6) are listed as IV estimation results. The DWH exogenous test refers to the Durbin–Wu–Hausman test, and it is mainly to test whether there is endogeneity between financial literacy and financial behaviors. The first-stage estimation of the F value and instrumental variable T value is mainly to test whether the instrumental variables we select are weak. Standard errors are single-clustered by community level and Z-statistics, which are shown in parentheses. Variable definitions are presented in Supplementary Table 1. *, **, and *** indicate significance at the 10, 5, and 1% levels, respectively.

Can limited attention act as an underlying behavioral mechanism through which financial literacy influences financial behaviors in the long term? In the previous two sections, we analyzed the impact of financial literacy on limited attention and the impact of limited attention on financial behavior. In fact, the analysis of these two parts is the mediation effect model. By combining the estimation results in Tables 5, 6, we find that financial literacy has current, long-term, and dynamic effects on residents’ limited attention. Moreover, limited attention has current and long-term effects on other financial behaviors except for social medical insurance. Financial behaviors (such as having a business bank account; participating in the stock market and venture capital; having social health insurance, commercial insurance, and pension plans; and holding a credit card) have dynamic effects. The results show that the impact of financial literacy on financial behavior is mainly achieved through the channel that affects residents’ limited attention. Interestingly, the limited attention to the current and long-term effects on social health insurance is insignificant. At the same time, financial literacy has no significant effect on social medical insurance’s long-term and dynamic effects. However, improving residents’ limited attention can improve residents’ social medical insurance decision-making. It further illustrates that the impact of financial literacy on social health insurance can be channeled through raising limited attention from residents.

### Robustness Test

To ensure that the estimated results of this study are robust, we conducted extensive robustness tests. Specifically, our robustness test included using sub-indices of financial literacy, using factor analysis to measure the level of financial literacy of residents, excluding households working in the financial industry, adding community peer effect as control variables, and replacing new models (such as using the OLS model and the Poisson model). We performed the propensity score matching method (PSM) to verify the robustness of the effect of limited attention on financial behavior.

First, we used the answers that we obtained for each subitem of financial literacy. The respondents’ answers to each question showed different levels of financial literacy (Lusardi and Mitchell, 2014). Lusardi and Mitchell (2014) believed that the financial literacy exhibited by respondents’ answers to each question varies. Therefore, the impact of respondents’ correct answers on interest rate calculation, inflation, and venture capital investment on financial behavior decisions must be estimated. A total of three dummy variables, namely, the correct answers to interest rate (*FL1*) calculation questions, inflation questions (*FL2*), and venture capital questions (*FL3*), were selected to investigate their impact on their financial behaviors. The robustness test results are shown in Table 8. The estimated results were consistent with the benchmark regression results.

**TABLE 8:** Robust test by considering subitem of financial literacy.

Variable	(1)	(2)	(3)	(4)	(5)	(6)	(7)	(8)
	
	BA	SM	VM	SMI	CHI	PP	CC	LA
**Panel A: Current effect**								
FL1	0.0407*** (7.1957)	0.0311*** (6.6478)	0.0335*** (7.0232)	0.0034 (0.8343)	0.0149*** (3.2609)	0.0205*** (3.4569)	0.0186*** (3.3256)	0.0640*** (9.2947)
FL2	0.0085 (1.2107)	0.0223*** (4.2003)	0.0204*** (3.6165)	0.0034 (0.7234)	−0.0044 (−0.8048)	0.0022 (0.3303)	0.0158** (2.3226)	0.0764*** (9.4628)
FL3	0.0192*** (2.6322)	−0.0043 (−0.6320)	0.0179** (2.3841)	0.0066 (1.2672)	0.0257*** (3.7606)	−0.0038 (−0.4442)	0.0426*** (5.2364)	0.0744*** (7.7420)
Controls	Yes	Yes	Yes	Yes	Yes	Yes	Yes	Yes
Province dummies	Yes	Yes	Yes	Yes	Yes	Yes	Yes	Yes
*N*	20,557	20,557	20,557	20,557	20,557	20,557	20,557	20,557
Pseudo *R*^2^	0.1289	0.2697	0.2705	0.0526	0.1107	0.1306	0.2438	0.1069
**Panel B: Long-term effect**								
FL1	0.0119*** (2.6360)	0.0236*** (4.6066)	0.0292*** (5.3943)	0.0023 (0.5055)	0.0205*** (4.2037)	0.0511*** (8.0388)	0.0214*** (2.9879)	0.0498*** (6.1282)
FL2	−0.0036 (−0.6747)	0.0284*** (4.6233)	0.0298*** (4.5163)	−0.0004 (−0.0859)	0.0064 (1.0900)	0.0767*** (9.8933)	0.0037 (0.4655)	0.0182* (1.8151)
FL3	0.0059 (1.1087)	−0.0149** (−2.0858)	−0.0020 (−0.2534)	0.0006 (0.1189)	0.0325*** (4.0489)	−0.0058 (−0.7398)	0.0283*** (2.9655)	0.0434*** (3.9421)
Controls	Yes	Yes	Yes	Yes	Yes	Yes	Yes	Yes
Province dummies	Yes	Yes	Yes	Yes	Yes	Yes	Yes	Yes
*N*	13,869	13,869	13,869	13,869	13,869	13,869	13,869	13,869
Pseudo *R*^2^	0.0792	0.2290	0.2355	0.0225	0.0852	0.1371	0.1944	0.0754
**Panel C: Dynamic effect**								
FL1	0.1153*** (4.4512)	−0.0250 (−0.7815)	−0.0246 (−0.8332)	−0.0004 (−0.0122)	−0.0189 (−0.6713)	0.0953*** (3.5402)	−0.0057 (−0.2160)	0.0663*** (3.0079)
FL2	0.0494 (1.3914)	0.0652 (1.3161)	0.0884* (1.9468)	−0.0101 (−0.2465)	0.0970** (2.3525)	0.2750*** (7.8896)	0.0200 (0.5421)	0.0038 (0.1209)
FL3	0.0579* (1.6790)	−0.0174 (−0.5175)	0.0513* (1.6579)	−0.0370 (−0.9908)	0.0511* (1.6527)	−0.0091 (−0.2776)	0.0046 (0.1667)	0.0487* (1.9506)
Controls	Yes	Yes	Yes	Yes	Yes	Yes	Yes	Yes
Province dummies	Yes	Yes	Yes	Yes	Yes	Yes	Yes	Yes
*N*	13,065	13,065	13,065	13,065	13,065	13,065	13,065	13,065
Pseudo *R*^2^	0.0476	0.0216	0.0175	0.0061	0.0099	0.0227	0.0040	0.0067

This table presents regression results for the robust test by considering the subitem of financial literacy. The dependent variable is shown above each column. Controls include GENDER, AGE, AGE2, EDU, MAR, HEALTH, FL, OLD, CHILD, FS, RL, RA, HO, OH, TA, TI. Standard errors are single-clustered by community level and Z-statistics, which are shown in parentheses. Variable definitions are presented in Supplementary Table 1. *, **, and *** indicate significance at the 10, 5, and 1% levels, respectively.

Second, measuring respondents’ objective financial literacy through factor analysis has also been used in many kinds of literature (Lusardi and Mitchell, 2010; Xu et al., 2021). The function of factor analysis is mainly used to classify various factors and reduce the dimensionality of the variables to be measured (Thurstone, 1931). To this end, we used the three questions on interest rate calculation, inflation calculation, and venture capital to construct two types of dummy variables: whether the answer was correct, the answer was unknown, or the answer could not be calculated. Therefore, according to the three questions, six variables could be constructed by the iterative principal factor method for factor analysis. We selected factors with eigenvalues greater than or equal to 1 as the financial literacy level of respondents and defined it as *Literacy*. Table 9 reports the current, long-term, and dynamic effects of using factor analysis to measure financial literacy on households’ financial behavior and limited attention. These results are consistent with our benchmark regression results.

**TABLE 9:** Robust test by considering proxy indicators of financial literacy.

Variable	(1)	(2)	(3)	(4)	(5)	(6)	(7)	(8)
	
	BA	SM	VM	SMI	CHI	PP	CC	LA
**Panel A: Current effect**								
Literacy	0.0094*** (3.0657)	0.0118*** (3.8024)	0.0232*** (6.7368)	0.0119* (1.8406)	0.0173*** (5.3138)	0.0107** (2.1927)	0.0273*** (6.9432)	0.0480*** (11.7331)
Controls	Yes	Yes	Yes	Yes	Yes	Yes	Yes	Yes
Province dummies	Yes	Yes	Yes	Yes	Yes	Yes	Yes	Yes
*N*	20,577	20,577	20,577	20,577	20,577	20,577	20,577	20,577
Pseudo *R*^2^	0.1284	0.2678	0.2704	0.0525	0.1115	0.1302	0.2462	0.1120
**Panel B: Long-term effect**								
Literacy	0.0033** (2.5188)	0.0017** (2.4912)	0.0075** (2.0422)	0.0014 (0.6353)	0.0130*** (3.4118)	0.0056* (1.6461)	0.0160*** (3.7742)	0.0239*** (5.1420)
Controls	Yes	Yes	Yes	Yes	Yes	Yes	Yes	Yes
Province dummies	Yes	Yes	Yes	Yes	Yes	Yes	Yes	Yes
*N*	13,869	13,869	13,869	13,869	13,869	13,869	13,869	13,869
Pseudo *R*^2^	0.0795	0.2250	0.2319	0.0226	0.0823	0.1189	0.1943	0.0749
**Panel C: Dynamic effect**								
Literacy	0.0237* (1.8601)	0.0104* (1.9458)	0.0427** (2.0419)	0.0168 (1.0703)	0.0012** (2.1124)	0.0198** (2.4294)	0.0011** (2.1087)	0.0378*** (3.8018)
Controls	Yes	Yes	Yes	Yes	Yes	Yes	Yes	Yes
Province dummies	Yes	Yes	Yes	Yes	Yes	Yes	Yes	Yes
*N*	13,869	13,869	13,869	13,869	13,869	13,869	13,869	13,869
Pseudo *R*^2^	0.0463	0.0214	0.2319	0.0161	0.0092	0.0164	0.0140	0.0066

This table presents regression results for the robust test by considering proxy indicators of financial literacy. The dependent variable is shown above each column. Controls include GENDER, AGE, AGE2, EDU, MAR, HEALTH, FL, OLD, CHILD, FS, RL, RA, HO, OH, TA, TI. Standard errors are single-clustered by community level and Z-statistics, which are shown in parentheses. Variable definitions are presented in Supplementary Table 1. *, **, and *** indicate significance at the 10, 5, and 1% levels, respectively.

Third, the respondents who were engaged in the financial industries often had rich financial literacy. Hence, they might differ from ordinary households in terms of financial literacy and household income levels. Those individuals who worked in financial industries must be excluded. The results showed that the current, long-term, and dynamic effects of financial literacy on financial behaviors were still consistent. The results are presented in Table 10. Financial literacy has current effects on all financial behaviors and long-term and dynamic effects on other financial behaviors except social health insurance. Meanwhile, financial literacy has current, long-term, and dynamic effects on residents’ limited attention.

**TABLE 10:** Robust test by excluding those individuals who worked in financial industries.

Variable	(1)	(2)	(3)	(4)	(5)	(6)	(7)	(8)
	
	BA	SM	VM	SMI	CHI	PP	CC	LA
**Panel A: Current effect**								
FL	0.0252*** (7.2270)	0.0213*** (7.2893)	0.0258*** (8.4471)	0.0044* (1.7612)	0.0102*** (3.7669)	0.0086** (2.3798)	0.0218*** (6.3539)	0.0698*** (16.3276)
Controls	Yes	Yes	Yes	Yes	Yes	Yes	Yes	Yes
Province dummies	Yes	Yes	Yes	Yes	Yes	Yes	Yes	Yes
*N*	20,201	20,201	20,201	20,201	20,201	20,201	20,201	20,201
Pseudo *R*^2^	0.1275	0.2656	0.2663	0.0524	0.1077	0.1306	0.2357	0.1006
**Panel B: Long-term effect**								
FL	0.0058** (2.0865)	0.0177*** (5.2710)	0.0228*** (6.6529)	0.0009 (0.3610)	0.0129** (2.0912)	0.0418*** (12.4580)	0.0165*** (3.9904)	0.0378*** (7.4669)
Controls	Yes	Yes	Yes	Yes	Yes	Yes	Yes	Yes
Province dummies	Yes	Yes	Yes	Yes	Yes	Yes	Yes	Yes
*N*	13,674	13,674	13,674	13,674	13,674	13,674	13,674	13,674
Pseudo *R*^2^	0.0767	0.2240	0.2306	0.0220	0.1230	0.1297	0.1878	0.0709
**Panel C: Dynamic effect**								
FL	0.0571*** (3.7425)	0.0015** (2.0746)	0.0051** (2.2777)	−0.0134 (−0.7430)	0.0312* (1.9011)	0.1159*** (7.7589)	0.0029** (2.1930)	0.0455*** (3.5540)
Controls	Yes	Yes	Yes	Yes	Yes	Yes	Yes	Yes
Province dummies	Yes	Yes	Yes	Yes	Yes	Yes	Yes	Yes
*N*	13,674	13,674	13,674	13,674	13,674	13,674	13,674	13,674
Pseudo *R*^2^	0.0468	0.0209	0.0170	0.0060	0.0094	0.0204	0.0038	0.0068

This table presents regression results for the robust test by excluding those individuals who worked in financial industries. The dependent variable is shown above each column. Controls include GENDER, AGE, AGE2, EDU, MAR, HEALTH, FL, OLD, CHILD, FS, RL, RA, HO, OH, TA, TI. Standard errors are single-clustered by community level and Z-statistics, which are shown in parentheses. Variable definitions are presented in Supplementary Table 1. *, **, and *** indicate significance at the 10, 5, and 1% levels, respectively.

Fourth, some literature studies have shown that peer effect will affect individual investment behavior (Bursztyn et al., 2014; Ouimet and Tate, 2020). The community cohort effect in the model must be controlled. Therefore, when discussing the impact of financial literacy on financial behavior, we included the community average (peer effect) of that financial behavior. We defined the newly generated peer effect variables as *BA_*c*_, SM_*c*_, VM_*c*_, SMIv, CHI_*c*_, PP_*c*_*, and *CC*_*c*_. When exploring the relationship between financial literacy and limited attention, we defined the newly generated peer effect variables as *LA*_*c*_. After adding the peer effect control variable, the estimated results are reported in Table 11. A peer effect was observed in financial behavior decision-making. After controlling for this variable, the impact of financial literacy on financial behavior and limited attention was still consistent with the benchmark regression results.

**TABLE 11:** Robust test by controlling the effect of community peer effect.

Variable	(1)	(2)	(3)	(4)	(5)	(6)	(7)	(8)
	
	BA	SM	VM	SMI	CHI	PP	CC	LA
**Panel A: Current effect**								
FL	0.0167*** (6.1970)	0.0147*** (5.4862)	0.0182*** (6.5044)	0.0043** (2.0783)	0.0064*** (2.5970)	0.0043** (2.4943)	0.0165*** (5.1456)	0.0399*** (9.9458)
BA_*c*_	0.6319*** (73.9930)							
SM_*c*_		0.4953*** (47.8212)						
VM_*c*_			0.5162*** (51.7014)					
SMI_*c*_				0.5841*** (48.8075)				
CHI_*c*_					0.6574*** (46.4209)			
PP_*c*_						0.6693*** (79.9465)		
CC_*c*_							0.5710*** (54.0709)	
LA_*c*_								0.7945*** (81.6361)
Controls	Yes	Yes	Yes	Yes	Yes	Yes	Yes	Yes
Province dummies	Yes	Yes	Yes	Yes	Yes	Yes	Yes	Yes
N	20,577	20,577	20,577	20,577	20,577	20,577	20,577	20,577
Pseudo R^2^	0.2633	0.3613	0.3550	0.2185	0.2237	0.2792	0.3202	0.1911
**Panel B: Long-term effect**								
LA	0.0046* (1.7304)	0.0139*** (4.2538)	0.0185*** (5.5356)	0.0010 (0.4043)	0.0161*** (5.3000)	0.0411*** (13.2918)	0.0143*** (3.4970)	0.0301*** (5.7870)
BA_*c*_	0.0645*** (6.0944)							
SM_*c*_		0.3401*** (25.0850)						
VM_*c*_			0.3485*** (24.6074)					
SMI_*c*_				0.1614*** (8.3887)				
CHI_*c*_					0.2704*** (13.9389)			
PP_*c*_						0.2927*** (21.5132)		
CC_*c*_							0.3005*** (16.8265)	
LA_*c*_								0.1967*** (9.0152)
Controls	Yes	Yes	Yes	Yes	Yes	Yes	Yes	Yes
Province dummies	Yes	Yes	Yes	Yes	Yes	Yes	Yes	Yes
*N*	13,869	13,869	13,869	13,869	13,869	13,869	13,869	13,869
Pseudo *R*^2^	0.0851	0.2849	0.2867	0.0364	0.1090	0.1843	0.2118	0.0805
**Panel C: Dynamic effect**								
LA	0.0150*** (3.9373)	0.0074*** (2.6980)	0.0105*** (3.3429)	−0.0021 (−0.6597)	0.0090*** (2.7921)	0.0004** (2.1284)	0.0016** (2.4058)	0.0192** (2.5339)
BA_*c*_	−0.5606*** (−38.6560)							
SM_*c*_		0.2125*** (12.6056)						
VM_*c*_			0.2402*** (14.0063)					
SMI_*c*_				−0.4992*** (−25.0378)				
CHI_*c*_					0.4128*** (19.6443)			
PP_*c*_						−0.3358*** (−22.8102)		
CC_*c*_							0.0642*** (3.2725)	
LA_*c*_								1.2382*** (25.5987)
Controls	Yes	Yes	Yes	Yes	Yes	Yes	Yes	Yes
Province dummies	Yes	Yes	Yes	Yes	Yes	Yes	Yes	Yes
*N*	13,869	13,869	13,869	13,869	13,869	13,869	13,869	13,869
Pseudo *R*^2^	0.1884	0.1525	0.1441	0.0947	0.1107	0.1082	0.0372	0.0287

This table presents regression results for the robust test by controlling the community peer effect. The dependent variable is shown above each column. Controls include GENDER, AGE, AGE2, EDU, MAR, HEALTH, FL, OLD, CHILD, FS, RL, RA, HO, OH, TA, TI. Standard errors are single-clustered by community level and Z-statistics, which are shown in parentheses. Variable definitions are presented in Supplementary Table 1. *, **, and *** indicate significance at the 10, 5, and 1% levels, respectively.

Fifth, we employed an OLS model to re-estimate the impact of financial literacy on financial behavior and limited attention. The estimated results are listed in Table 12. The results in Table 12 show that the current, long-term, and dynamic effects of financial literacy on financial behavior and limited attention remain significant. However, its estimated coefficients have changed. This result is the same as what we estimated using the Probit model.

**TABLE 12:** Robust test by estimation using the OLS model.

Variable	(1)	(2)	(3)	(4)	(5)	(6)	(7)	(8)
	
	BA	SM	VM	SMI	CHI	PP	CC	LA
**Panel A: Current effect**								
FL	0.0250*** (7.2962)	0.0202*** (6.2112)	0.0244*** (7.3304)	0.0040* (1.6485)	0.0082*** (2.9900)	0.0077** (2.1668)	0.0191*** (5.3832)	0.0563*** (12.7404)
Controls	Yes	Yes	Yes	Yes	Yes	Yes	Yes	Yes
Province dummies	Yes	Yes	Yes	Yes	Yes	Yes	Yes	Yes
*N*	20,577	20,577	20,577	20,577	20,577	20,577	20,577	20,577
*R* ^2^	0.1214	0.2153	0.2356	0.0320	0.0673	0.1259	0.2493	0.1453
**Panel B: Long-term effect**								
FL	0.0061** (2.2018)	0.0180*** (4.8191)	0.0238*** (6.3057)	0.0010 (0.3868)	0.0172*** (5.4548)	0.0385*** (12.4869)	0.0153*** (3.6790)	0.0338*** (6.4434)
Controls	Yes	Yes	Yes	Yes	Yes	Yes	Yes	Yes
Province dummies	Yes	Yes	Yes	Yes	Yes	Yes	Yes	Yes
*N*	13,869	13,869	13,869	13,869	13,869	13,869	13,869	13,869
*R* ^2^	0.0364	0.1582	0.1798	0.0104	0.0496	0.0884	0.2067	0.0987
**Panel C: Dynamic effect**								
FL	0.0177*** (3.8051)	0.0014** (2.0996)	0.0019** (2.2357)	−0.0026 (−0.7448)	0.0068* (1.8341)	0.0336*** (7.8148)	0.0013** (2.2670)	0.0234*** (3.5679)
Controls	Yes	Yes	Yes	Yes	Yes	Yes	Yes	Yes
Province dummies	Yes	Yes	Yes	Yes	Yes	Yes	Yes	Yes
*N*	13,869	13,869	13,869	13,869	13,869	13,869	13,869	13,869
*R* ^2^	0.0606	0.0168	0.0158	0.0049	0.0087	0.0241	0.0045	0.0119

This table presents regression results for the robust test by estimation using the OLS model. The dependent variable is shown above each column. Controls include GENDER, AGE, AGE2, EDU, MAR, HEALTH, FL, OLD, CHILD, FS, RL, RA, HO, OH, TA, TI. Standard errors are single-clustered by community level and T-statistics, which are shown in parentheses. Variable definitions are presented in Supplementary Table 1. *, **, and *** indicate significance at the 10, 5, and 1% levels, respectively.

Sixth, we employed a Poisson model to re-estimate the impact of financial literacy on financial behavior and limited attention. The estimated results are listed in Table 13. The results in Table 13 show that the current and long-term effects of financial literacy on financial behavior and limited attention remain significant. However, its estimated coefficients have changed. Considering that Poisson estimation is only applicable to the case where the dependent variable is greater than or equal to 0, we could not test the dynamic effect of financial literacy. From the current estimation results, our benchmark regression results are still supported.

**TABLE 13:** Robust test by estimation using the Poisson model.

Variable	(1)	(2)	(3)	(4)	(5)	(6)	(7)	(8)
	
	BA	SM	VM	SMI	CHI	PP	CC	LA
**Panel A: Current effect**								
FL	0.0309*** (7.3066)	0.1289*** (6.8416)	0.1302*** (8.0253)	0.0043 (1.6355)	0.0831*** (3.6706)	0.0094** (2.1193)	0.0783*** (5.9733)	0.1215*** (12.3170)
Controls	Yes	Yes	Yes	Yes	Yes	Yes	Yes	Yes
Province dummies	Yes	Yes	Yes	Yes	Yes	Yes	Yes	Yes
*N*	20,577	20,577	20,577	20,577	20,577	20,577	20,577	20,577
Pseudo *R*^2^	0.0115	0.1898	0.1805	0.0013	0.0932	0.0132	0.1474	0.0498
**Panel B: Long-term effect**								
FL	0.0066** (2.2198)	0.1524*** (5.1912)	0.1687*** (6.7929)	0.0011 (0.3893)	0.1910*** (5.8015)	0.0439*** (12.2390)	0.0677*** (4.3832)	0.0850*** (6.5444)
Controls	Yes	Yes	Yes	Yes	Yes	Yes	Yes	Yes
Province dummies	Yes	Yes	Yes	Yes	Yes	Yes	Yes	Yes
*N*	13,869	13,869	13,869	13,869	13,869	13,869	13,869	13,869
Pseudo *R*^2^	0.0011	0.1775	0.1761	0.0003	0.0747	0.0057	0.1220	0.0377

This table presents regression results for the robust test by estimation using the Poisson model. Poisson estimators require values of the dependent variable to be greater than 0, so dynamic effects cannot be used with this model. The dependent variable is shown above each column. Controls include GENDER, AGE, AGE2, EDU, MAR, HEALTH, FL, OLD, CHILD, FS, RL, RA, HO, OH, TA, TI. Standard errors are single-clustered by community level and Z-statistics, which are shown in parentheses. Variable definitions are presented in Supplementary Table 1. *, **, and *** indicate significance at the 10, 5, and 1% levels, respectively.

Seventh, the residents’ attention to economic and financial-related information may be a rational self-selection behavior. PSM was adopted to consider potential selection bias. A total of three matching methods, namely, radius matching, nearest-neighbor matching, and kernel matching, were used to test the robustness. Comparing the results above, Table 14 shows the ATT estimation of the three matching methods. The results are consistent with the estimated results in Table 6. Compared with residents who ignored the impact of economic and financial information on financial behavior decision-making, those with active concern about relative information are associated with a significantly higher participation rate in the financial market and some financial services (e.g., holding credit cards) in the current and long term. Increasing residents’ limited attention can promote household financial market participation and purchase-related financial products and services and improve financial behavior.

**TABLE 14:** Robust test by PSM.

Variable	Radius match (*r* = 0.01)			Nearest neighbor match (1:1)			Nuclear match		
	ATT	Standard error	*T* value	ATT	Standard error	*T* value	ATT	Standard error	*T* value
**Panel A: Current effect**									
BA	0.8718	0.007	3.12***	0.026	0.008	3.22***	0.8719	0.007	3.40***
SM	0.2839	0.006	23.47***	0.154	0.010	15.26***	0.2849	0.006	24.34***
VM	0.3301	0.006	22.79***	0.160	0.010	15.66***	0.3310	0.006	23.74***
SMI	0.9390	0.005	6.79***	0.023	0.007	2.51**	0.9290	0.005	5.92***
CHI	0.1590	0.006	6.59***	0.038	0.009	4.46***	0.1593	0.006	6.99***
PP	0.8374	0.007	2.88***	0.019	0.008	2.35**	0.8276	0.007	3.17***
CC	0.3781	0.008	7.92***	0.067	0.011	6.04***	0.3788	0.007	8.79***
**Panel B: Long-term effect**									
BA	0.9728	0.0045	6.96***	0.029	0.006	4.76***	0.9727	0.0044	7.14***
SM	0.2278	0.0065	19.44***	0.136	0.010	13.16***	0.2281	0.0064	20.19***
VM	0.2637	0.0069	19.68***	0.149	0.010	14.99***	0.2641	0.0069	20.43***
SMI	0.9501	0.0046	1.89*	0.005	0.006	0.81	0.9501	0.0045	1.98**
CHI	0.1357	0.0058	7.20***	0.041	0.009	4.40***	0.1360	0.0058	7.54***
PP	0.8976	0.0066	3.36***	0.010	0.009	1.14	0.8977	0.0065	3.62***
CC	0.3862	0.0086	12.52***	0.092	0.013	7.19***	0.3862	0.0085	13.04***
**Panel C: Dynamic effect**									
BA	0.0925	0.0091	2.28**	0.023	0.011	2.07**	0.0926	0.0088	2.41**
SM	0.0534	0.0060	5.50***	0.043	0.011	4.09***	0.0532	0.0059	5.79***
VM	0.0662	0.0068	5.71***	0.047	0.011	4.36***	0.0659	0.0067	5.95***
SMI	0.0017	0.0066	1.61	−0.010	0.008	−1.19	0.0017	0.0064	−1.60
CHI	0.0370	0.0072	1.92*	0.007	0.010	0.65	0.0368	0.0070	1.88*
PP	0.0474	0.0086	2.30**	0.029	0.011	2.75***	0.4748	0.0083	2.52**
CC	0.0462	0.0088	2.25**	0.037	0.014	2.02**	0.0380	0.0086	2.02**

Figures in brackets are heteroskedasticity-consistent t-statistics. *, **, and *** indicate significance at the 10, 5, and 1% levels, respectively.

## Heterogeneity Analysis

In this section, we mainly explore whether the impact of financial literacy on financial behavior is heterogeneous between urban and rural households, different genders, and various education levels. We continue to use models (1–4) and perform heterogeneity analysis using a subsampled approach.

### Urban and Rural Differences

The existence of China’s dual economic structure may create significant differences in the economic environment between urban and rural areas (Zhang, 2019). Therefore, residents from urban or rural areas may have diversified financial literacy. We further split our sample into two groups (urban residents vs. rural residents) and tested whether the impact of financial literacy on financial behavior was robust in urban or rural families. The estimated results are listed in Table 15. Panels A, B, and C reported the results of the current, long-term, and dynamic impacts.

**TABLE 15:** Urban and rural heterogeneity analysis.

Variable	(1)	(2)	(3)	(4)	(5)	(6)	(7)
	
	BA	SM	VM	SMI	CHI	PP	CC
**Panel A: Current effect**							
**Urban household**							
FL	0.0223*** (6.3544)	0.0206*** (5.5482)	0.0258*** (6.6850)	0.0037** (2.2886)	0.0101*** (3.0671)	0.0106*** (2.7985)	0.0166*** (3.9261)
Controls	Yes	Yes	Yes	Yes	Yes	Yes	Yes
Province dummies	Yes	Yes	Yes	Yes	Yes	Yes	Yes
*N*	16,366	16,366	16,366	16,366	16,366	16,366	16,366
Pseudo *R*^2^	0.1008	0.2254	0.2271	0.0608	0.1021	0.1662	0.2253
**Rural household**							
FL	0.0066 (0.6625)	0.0062*** (2.8551)	0.0062** (2.4827)	0.0063 (1.1970)	0.0040 (0.8642)	−0.0081 (−0.8050)	0.0164*** (3.0445)
Controls	Yes	Yes	Yes	Yes	Yes	Yes	Yes
Province dummies	Yes	Yes	Yes	Yes	Yes	Yes	Yes
*N*	4,211	4,211	4,211	4,211	4,211	4,211	4,211
Pseudo *R*^2^	0.1078	0.3386	0.2503	0.0514	0.0950	0.0433	0.1430
**Panel B: Long-term effect**							
**Urban household**							
FL	0.0065** (2.2237)	0.0177*** (3.8385)	0.0248*** (5.2895)	0.0054* (1.6837)	0.0205*** (5.2967)	0.0507*** (14.1202)	0.0194*** (3.6885)
Controls	Yes	Yes	Yes	Yes	Yes	Yes	Yes
Province dummies	Yes	Yes	Yes	Yes	Yes	Yes	Yes
*N*	10,267	10,267	10,267	10,267	10,267	10,267	10,267
Pseudo *R*^2^	0.0752	0.1779	0.1814	0.0288	0.0835	0.1778	0.1853
**Rural household**							
FL	−0.0041 (−0.6066)	0.0062*** (2.7307)	0.0065*** (2.6481)	0.0111** (2.2203)	0.0124** (2.3771)	0.0093 (1.1101)	0.0073 (1.0839)
Controls	Yes	Yes	Yes	Yes	Yes	Yes	Yes
Province dummies	Yes	Yes	Yes	Yes	Yes	Yes	Yes
*N*	3,602	3,602	3,602	3,602	3,602	3,602	3,602
Pseudo *R*^2^	0.0675	0.2724	0.2573	0.0335	0.0510	0.0519	0.0847
**Panel C: Dynamic effect**							
**Urban household**							
FL	0.0660*** (3.7299)	0.0038** (2.1769)	0.0077** (2.3857)	0.0158 (0.7805)	0.0253 (1.3949)	0.1491*** (8.4575)	0.0101*** (2.5859)
Controls	Yes	Yes	Yes	Yes	Yes	Yes	Yes
Province dummies	Yes	Yes	Yes	Yes	Yes	Yes	Yes
*N*	10,267	10,267	10,267	10,267	10,267	10,267	10,267
Pseudo *R*^2^	0.0334	0.0192	0.0145	0.0087	0.0089	0.0283	0.0041
**Rural household**							
FL	−0.0308 (−1.0360)	0.0378 (0.5961)	0.0480 (0.8187)	0.1047*** (2.8589)	0.0461** (2.2241)	0.0605** (2.2334)	−0.0304 (−0.9343)
Controls	Yes	Yes	Yes	Yes	Yes	Yes	Yes
Province dummies	Yes	Yes	Yes	Yes	Yes	Yes	Yes
*N*	3,602	3,602	3,602	3,602	3,602	3,602	3,602
Pseudo *R*^2^	0.0437	0.0540	0.0480	0.0158	0.0128	0.0186	0.0121

This table presents regression results for the urban and rural heterogeneity analysis. The dependent variable is shown above each column. Controls include GENDER, AGE, AGE2, EDU, MAR, HEALTH, FL, OLD, CHILD, FS, RL, RA, HO, OH, TA, TI. Standard errors are single-clustered by community level and Z-statistics, which are shown in parentheses. Variable definitions are presented in Supplementary Table 1. *, **, and *** indicate significance at the 10, 5, and 1% levels, respectively.

Panel A shows that increased financial literacy helps urban households participate in financial markets, medical insurance, commercial insurance, and social pension insurance and hold credit cards and formal bank accounts. The impact of financial literacy on the financial behavior of rural households, except for participating in social pension insurance, social medical insurance, and commercial insurance, and financial literacy, is positively correlated with other financial behaviors. One possible reason is that with the development of new rural cooperative medical insurance in rural areas, local governments encourage farmers to participate in this kind of insurance, which has an existing substitution effect on social medical insurance and weakens the association between the financial literacy and participation of medical insurance in rural areas. Panel B results showed that financial literacy has a significant and positive impact on all financial behaviors of urban households in the long term. Financial literacy has a significant positive effect on all financial behaviors in rural areas, except holding formal bank accounts, holding credit cards, and participating in pension insurance. This phenomenon may be due to the low penetration rate of credit cards and pension insurance in rural areas and the lack of understanding of its role among residents. As rural households tend to save, formal bank accounts are held under policy conditions. Panel C results showed that financial literacy significantly improved some financial behaviors (e.g., holding formal accounts, being involved in the stock market and venture capital, participating in pension insurance, and holding credit cards) for urban households. Meanwhile, financial literacy also had a significant dynamic improvement effect on some financial behaviors (e.g., social insurance participation, commercial insurance participation, and pension participation) for rural households. The observed results were close to previous findings.

In addition, we found that the marginal impact of rural family financial literacy on having formal bank accounts, participating in the financial market, participating in pension plans, and participating in commercial insurance was less than that for urban families. Compared with rural areas with lower economic development levels and education levels, urban families participated in the financial market and had better health care. Therefore, families with higher financial literacy were more inclined to implement such financial behavior. Above all, financial literacy promotes families (in both urban and rural areas) to buy relevant financial products and services.

### Gender Difference

Coile and Milligan (2009) found that as women have a lower risk appetite than men and their investment behavior is relatively conservative, women are more inclined to hold bonds, whereas men are more inclined to participate in the stock market. Furthermore, Robb and Woodyard (2011) found that best financial practices are influenced by financial literacy and differ by gender. Therefore, we believe that gender differences are the factor in the impact of financial literacy on financial behavior. We further divided the sample into two groups (male vs. female) and tested whether the effect of financial literacy on financial behavior was strong in male or female households. The estimated results are listed in Table 16. Panels A–C report results for current, long-term, and dynamic effects. The results of panel A show that financial literacy has a significant effect on all financial behaviors of men. Conversely, it is not significant for women’s participation in social medical insurance, social pension insurance, and commercial insurance. Panel B results show that financial literacy has a significant long-term effect on all financial behaviors of men, except holding a formal bank account and participating in social medical insurance. Financial literacy had significant long-term effects on women’s financial behaviors, except for social medical insurance participation. Panel C results show that financial literacy can only improve two financial behaviors of men holding a formal bank account and participating in pension insurance. However, it can improve most women’s financial behaviors. The results in Table 16 show heterogeneity in the current, long-term, and dynamic effects of financial literacy on financial behavior.

**TABLE 16:** Gender heterogeneity analysis.

Variable	(1)	(2)	(3)	(4)	(5)	(6)	(7)
	
	BA	SM	VM	SMI	CHI	PP	CC
**Panel A: Current effect**							
**Male**							
FL	0.0226*** (5.6134)	0.0174*** (5.5205)	0.0226*** (6.7526)	0.0054* (1.8425)	0.0129*** (4.3168)	0.0101** (2.3036)	0.0250*** (6.4197)
Controls	Yes	Yes	Yes	Yes	Yes	Yes	Yes
Province dummies	Yes	Yes	Yes	Yes	Yes	Yes	Yes
*N*	14,980	14,980	14,980	14,980	14,980	14,980	14,980
Pseudo *R*^2^	0.1283	0.2779	0.2773	0.0452	0.1044	0.1088	0.2414
**Female**							
FL	0.0315*** (4.9762)	0.0319*** (5.1638)	0.0346*** (5.3365)	0.0012 (0.2562)	0.0023 (0.3908)	0.0031 (0.5226)	0.0148** (2.2087)
Controls	Yes	Yes	Yes	Yes	Yes	Yes	Yes
Province dummies	Yes	Yes	Yes	Yes	Yes	Yes	Yes
*N*	5,597	5,597	5,597	5,597	5,597	5,597	5,597
Pseudo *R*^2^	0.1333	0.2457	0.2498	0.0794	0.1156	0.2022	0.2451
**Panel B: Long-term effect**							
**Male**							
FL	0.0011 (0.3535)	0.0127*** (3.4901)	0.0161*** (4.2439)	−0.0013 (−0.4243)	0.0163*** (4.7021)	0.0386*** (9.3346)	0.0164*** (3.3309)
Controls	Yes	Yes	Yes	Yes	Yes	Yes	Yes
Province dummies	Yes	Yes	Yes	Yes	Yes	Yes	Yes
*N*	10,470	10,470	10,470	10,470	10,470	10,470	10,470
Pseudo *R*^2^	0.0784	0.2379	0.2418	0.0258	0.0824	0.1182	0.1963
**Female**							
FL	0.0136** (2.4734)	0.0243*** (3.1393)	0.0374*** (4.6039)	0.0072 (1.3120)	0.0224*** (3.2997)	0.0468*** (8.3340)	0.0154* (1.7747)
Controls	Yes	Yes	Yes	Yes	Yes	Yes	Yes
Province dummies	Yes	Yes	Yes	Yes	Yes	Yes	Yes
*N*	3,399	3,399	3,399	3,399	3,399	3,399	3,399
Pseudo *R*^2^	0.0962	0.1971	0.2114	0.0277	0.0940	0.1824	0.1930
**Panel C: Dynamic effect**							
**Male**							
FL	0.0597*** (3.3917)	−0.0032 (−0.1409)	−0.0209 (−0.9945)	−0.0283 (−1.3721)	0.0161 (0.8539)	0.1090*** (6.3772)	−0.0006 (−0.0322)
Controls	Yes	Yes	Yes	Yes	Yes	Yes	Yes
Province dummies	Yes	Yes	Yes	Yes	Yes	Yes	Yes
*N*	10,470	10,470	10,470	10,470	10,470	10,470	10,470
Pseudo *R*^2^	0.0466	0.0214	0.0172	0.0049	0.0090	0.0170	0.0045
**Female**							
FL	0.0512* (1.6878)	0.0117** (2.3223)	0.0441** (2.2924)	0.0350 (1.0048)	0.0651** (2.0149)	0.1535*** (5.3036)	0.0177* (1.6894)
Controls	Yes	Yes	Yes	Yes	Yes	Yes	Yes
Province dummies	Yes	Yes	Yes	Yes	Yes	Yes	Yes
*N*	3,399	3,399	3,399	3,399	3,399	3,399	3,399
Pseudo *R*^2^	0.0538	0.0254	0.0212	0.0210	0.0120	0.0402	0.0098

This table presents regression results for the gender heterogeneity analysis. The dependent variable is shown above each column. Controls include AGE, AGE2, EDU, MAR, HEALTH, FL, OLD, CHILD, FS, RL, RA, HO, OH, TA, TI. Standard errors are single-clustered by community level and Z-statistics, which are shown in parentheses. Variable definitions are presented in Supplementary Table 1. *, **, and *** indicate significance at the 10, 5, and 1% levels, respectively.

### Education Level Difference

Guiso et al. (2004) showed that education, as an important human capital, can reduce the negative effects of insufficient social capital, thereby increasing the possibility of households participating in the financial market. Guiso and Paiella (2008) found that education level has a significant positive correlation with stock market participation because a higher education level can enable investors to obtain lasting income and wealth accumulation and enhance individuals’ ability to acquire and process market information. Moreover, a high education level can deepen people’s understanding of financial markets, financial products, and financial services. We further divided the sample into two groups (high education vs. low education) and tested whether the effect of financial literacy on financial behavior was strong in high education or low education households. Specifically, we defined families with a post-education level or above as highly educated households; otherwise, they were defined as low education households. The estimated results are listed in Table 17. Panels A–C report results for current, long-term, and dynamic effects. We found that financial literacy has no significant effect on the social health insurance participation of households with high education levels nor does it significantly improve their behavior of holding formal bank accounts and participating in commercial insurance. Meanwhile, financial literacy does not have a significant current effect on the pension insurance participation of households with low education levels, and no significant long-term effect is observed on households holding formal bank accounts and participating in social health insurance. However, it has a significant dynamic impact on holding a formal bank account and participating in social medical insurance and pension insurance. All of them are significant at a level of more than 5%. The results in Table 17 show that the impact of financial literacy on financial behavior varies across education levels.

**TABLE 17:** Education level heterogeneity analysis.

Variable	(1)	(2)	(3)	(4)	(5)	(6)	(7)
	
	BA	SM	VM	SMI	CHI	PP	CC
**Panel A: Current effect**							
**High education household**							
FL	0.0126*** (2.6450)	0.0292*** (3.7049)	0.0305*** (3.8200)	0.0001 (0.0310)	0.0165** (2.3412)	0.0054** (2.0827)	0.0287*** (3.3474)
Controls	Yes	Yes	Yes	Yes	Yes	Yes	Yes
Province dummies	Yes	Yes	Yes	Yes	Yes	Yes	Yes
*N*	5,066	5,066	5,066	5,066	5,066	5,066	5,066
Pseudo *R*^2^	0.0422	0.1655	0.1596	0.0698	0.0776	0.1665	0.1639
**Low education household**							
FL	0.0223*** (5.0943)	0.0161*** (5.5476)	0.0212*** (6.7541)	0.0053* (1.7941)	0.0072** (2.5028)	0.0068 (1.4568)	0.0128*** (3.3951)
Controls	Yes	Yes	Yes	Yes	Yes	Yes	Yes
Province dummies	Yes	Yes	Yes	Yes	Yes	Yes	Yes
*N*	15,511	15,511	15,511	15,511	15,511	15,511	15,511
Pseudo *R*^2^	0.1217	0.2404	0.2382	0.0553	0.1020	0.1149	0.1632
**Panel B: Long-term effect**							
**High education household**							
FL	0.0059* (1.8057)	0.0346*** (3.2686)	0.0459*** (4.3656)	−0.0022 (−0.4337)	0.0230*** (2.6560)	0.0313*** (7.7411)	0.0389*** (3.4691)
Controls	Yes	Yes	Yes	Yes	Yes	Yes	Yes
Province dummies	Yes	Yes	Yes	Yes	Yes	Yes	Yes
*N*	2,810	2,810	2,810	2,810	2,810	2,810	2,810
Pseudo *R*^2^	0.0396	0.1238	0.1299	0.0554	0.0749	0.1376	0.1328
**Low education household**							
FL	0.0029 (0.8387)	0.0110*** (3.4689)	0.0152*** (4.3963)	0.0011 (0.3646)	0.0165*** (4.9996)	0.0419*** (9.7588)	0.0105** (2.2628)
Controls	Yes	Yes	Yes	Yes	Yes	Yes	Yes
Province dummies	Yes	Yes	Yes	Yes	Yes	Yes	Yes
*N*	11,059	11,059	11,059	11,059	11,059	11,059	11,059
Pseudo *R*^2^	0.0642	0.1941	0.1937	0.0196	0.0583	0.1073	0.1236
**Panel C: Dynamic effect**							
**High education household**							
FL	−0.0446 (−1.1741)	0.0037** (2.1141)	0.0241*** (2.7646)	0.0088 (0.2290)	−0.0061 (−0.2052)	0.1663*** (5.2762)	0.0142** (2.4895)
Controls	Yes	Yes	Yes	Yes	Yes	Yes	Yes
Province dummies	Yes	Yes	Yes	Yes	Yes	Yes	Yes
*N*	2,810	2,810	2,810	2,810	2,810	2,810	2,810
Pseudo *R*^2^	0.0245	0.0166	0.0106	0.0303	0.0135	0.0667	0.0081
**Low education household**							
FL	0.0617*** (3.6405)	−0.0083 (−0.3391)	−0.0237 (−1.0499)	−0.0226 (−1.1473)	0.0403** (2.0804)	0.1104*** (6.6578)	0.0039 (0.2199)
Controls	Yes	Yes	Yes	Yes	Yes	Yes	Yes
Province dummies	Yes	Yes	Yes	Yes	Yes	Yes	Yes
*N*	11,059	11,059	11,059	11,059	11,059	11,059	11,059
Pseudo *R*^2^	0.0462	0.0251	0.0204	0.0079	0.0129	0.0177	0.0049

This table presents regression results for the education level heterogeneity analysis. The dependent variable is shown above each column. Controls include GENDER, AGE, AGE2, MAR, HEALTH, FL, OLD, CHILD, FS, RL, RA, HO, OH, TA, TI. Standard errors are single-clustered by community level and Z-statistics, which are shown in parentheses. Variable definitions are presented in Supplementary Table 1. *, **, and *** indicate significance at the 10, 5, and 1% levels, respectively.

## Conclusion

Using China Household Financial Survey Data (CHFS) of 2015 and 2017, we investigate the current, long-term, and dynamic influences of financial literacy on residents’ financial behavior and a potential mechanism between them. First, higher financial literacy of households is associated with active financial behaviors, such as current, long-term, and dynamic effects on participating in the stock market, participating in the risk market, having formal bank accounts, holding credit cards, purchasing medical insurance, and having pension insurance. Second, we found that financial literacy positively impacts residents’ current, long-term, and dynamic limited attention. Limited attention also has current, long-term, and dynamic effects on financial behavior. We reveal that limited attention acts as an underlying behavioral mechanism through which financial literacy influences current and long-term financial behaviors. Financial literacy also improves residents’ financial behavior through limited attention channels to develop in a more reasonable direction. The above conclusions still hold after a series of robustness tests. Some financial information (e.g., earnings announcements) is relatively less accessible to the public due to information asymmetry and high processing costs relative to media coverage (Oliver et al., 2022). In this regard, interpreting some information in making financial decisions is challenging for individuals who lack the relative financial literacy. In addition, the heterogeneity analysis found that the current, long-term, and dynamic effects of financial literacy on financial behavior were significantly different between urban and rural areas, between different genders, and at different education levels.

To enhance residents’ attention to economic and financial information, governments and financial institutions should strengthen long-term financial training in rural areas to encourage people to participate in some financial training projects (Sayinzoga et al., 2016). We should vigorously popularize financial literacy; strengthen the education and training of financial market investment, insurance, lending, and other financial literacy faced by residents in their daily lives; and improve their financial literacy. Specifically, we should be fully aware of the crucial role of financial literacy in advancing poverty reduction and the severe shortage of financial literacy for the individual in emerging countries. Financial education should be included into early childhood education to help children develop their financial literacy and cognitive ability. Financial education must be integrated into general school curricula. Any attempts to assess, comprehend, and enhance the financial literacy of households should be conducted with the strong support of governments and financial institutions.

## Data Availability Statement

The data that support the findings of this study are available from the China Household Finance Survey (CHFS) (https://chfser.swufe.edu.cn/datas/) conducted by the Survey and Research Center for China Household Finance at the Southwestern University of Finance and Economics (SWUFE), China.

## Author Contributions

All authors listed have made a substantial, direct, and intellectual contribution to the work, and approved it for publication.

## Conflict of Interest

The authors declare that the research was conducted in the absence of any commercial or financial relationships that could be construed as a potential conflict of interest.

## Publisher’s Note

All claims expressed in this article are solely those of the authors and do not necessarily represent those of their affiliated organizations, or those of the publisher, the editors and the reviewers. Any product that may be evaluated in this article, or claim that may be made by its manufacturer, is not guaranteed or endorsed by the publisher.

## References

[B1] Aboody D. Lehavy R. Trueman B. (2009). Limited attention and the earnings announcement returns of past stock market winners. *Rev. Account. Stud.* 15 317–344. 10.1007/s11142-009-9104-9

[B2] Abreu M. Mendes V. (2010). Financial literacy and portfolio diversification. *Quant. Financ.* 10 515–528. 10.1080/14697680902878105

[B3] Agnew J. R. Szykman L. R. (2005). Asset allocation and information overload: the influence of information display, asset choice, and investor experience. *J. Behav. Financ.* 6 57–70. 10.1207/s15427579jpfm0602_2

[B4] Aren S. Zengin A. N. (2016). Influence of financial literacy and risk perception on choice of investment. *Procedia–Soci. Behav. Sc.* 235 656–663. 10.1016/j.sbspro.2016.11.047

[B5] Barber B. M. Odean T. (2008). All that glitters: The effect of attention and news on the buying behavior of individual and institutional investors. *Rev. Financ. Stud.* 21 785–818. 10.1093/rfs/hhm079

[B6] Białowolski P. (2019). Economic sentiment as a driver for household financial behavior. *J. Behav. Exp. Econ.* 80 59–66. 10.1016/j.socec.2019.03.006

[B7] Bianchi M. (2018). Financial literacy and portfolio dynamics. *J. Financ.* 73 831–859. 10.1111/jofi.12605

[B8] Blagoeva R. R. Mom T. J. M. Jansen J. J. P. George G. (2020). Problem-solving or self-enhancement? A power perspective on how CEOs affect R&D search in the face of inconsistent feedback. *Acad. Manage. J.* 63 332–355. 10.5465/amj.2017.0999

[B9] Bottazzi L. Lusardi A. (2021). Stereotypes in financial literacy: evidence from PISA. *J. Corp. Financ* 71:101831. 10.1016/j.jcorpfin.2020.101831

[B10] Bursztyn L. Ederer F. Ferman B. Yuchtman N. (2014). Understanding mechanisms underlying peer effects: evidence from a field experiment on financial decisions. *Econometrica* 82 1273–1301. 10.3982/ECTA11991 30516143

[B11] Choi S. Choi W. Y. (2019). Effects of limited attention on investors’ trading behavior: evidence from online ranking data. *Pac-Basin. Financ. J.* 56 273–289. 10.1016/j.pacfin.2019.06.007

[B12] Clark R. Lusardi A. Mitchell O. S. (2017). Employee financial literacy and retirement plan behavior: a case study. *Econ. Inq.* 55 248–259. 10.1111/ecin.12389

[B13] Coile C. Milligan K. (2009). How household portfolios evolve after retirement: the effect of aging and health shocks. *Rev. Income. Wealth.* 55 226–248. 10.1111/j.1475-4991.2009.00320.x

[B14] Cordero J. M. Gil-Izquierdo M. Pedraja-Chaparro F. (2020). Financial education and student financial literacy: a cross-country analysis using PISA 2012 data. *Soc. Sci. J.* 59 15–33. 10.1016/j.soscij.2019.07.011

[B15] Falkinger J. (2008). Limited attention as a scarce resource in information-rich economies. *Econ. J.* 118 1596–1620. 10.1111/j.1468-0297.2008.02182.x

[B16] Guiso L. Paiella M. (2008). Risk aversion, wealth, and background risk. *J. Eur. Econ. Assoc.* 6 1109–1150. 10.1162/JEEA.2008.6.6.1109

[B17] Guiso L. Sapienza P. Zingales L. (2004). The role of social capital in financial development. *Am. Econ. Rev.* 94 526–556. 10.1257/0002828041464498

[B18] Habidin N. F. Zubir A. F. M. Fuzi N. M. Salleh M. I. (2020). The relationship between sustainable manufacturing practices, lean improvement and performance. *World. Rev. Entrep. Manag. Sustain. Dev.* 21 92–107. 10.1504/wremsd.2020.105529 35009967

[B19] Hill A. D. Johnson S. G. Greco L. M. O’Boyle E. H. Walter S. L. (2020). Endogeneity: A review and agenda for the methodology-practice divide affecting micro and macro research. *J. Manage.* 47 105–143. 10.1177/0149206320960533

[B20] Hsiao Y. J. Tsai W. C. (2018). Financial literacy and participation in the derivatives markets. *J. Bank. Financ.* 88 15–29. 10.1016/j.jbankfin.2017.11.006

[B21] Huston S. J. (2010). Measuring financial literacy. *J. Consum. Aff.* 44 296–316. 10.1111/j.1745-6606.2010.01170.x

[B22] Illeditsch P. K. Ganguli J. V. Condie S. (2021). Information inertia. *J. Financ.* 76 443–479. 10.1111/jofi.12979

[B23] Kahneman D. Tversky A. (1973). On the psychology of prediction. *Psychol. Rev.* 80 237–251. 10.1037/h0034747

[B24] Kaiser T. Lusardi A. Menkhoff L. Urban C. (2021). Financial education affects financial knowledge and downstream behaviors. *J. Financ. Econ* 10.1016/j.jfineco.2021.09.022 [Epub ahead of print].

[B25] Kim K. T. Lee J. (2017). Financial literacy and use of payday loans in the United States. *Appl. Econ. Lett.* 25 781–784. 10.1080/13504851.2017.1366635

[B26] Klapper L. Lusardi A. Panos G. A. (2012). Financial literacy and the financial crisis. *NBER Working Paper* 55:17930. 10.3386/w17930 34419315

[B27] Korkmaz A. G. Yin Z. Yue P. Zhou H. (2021). Does financial literacy alleviate risk attitude and risk behavior inconsistency? *Int. Rev. Econ. Financ.* 74 293–310. 10.1016/j.iref.2021.03.002

[B28] Lei X. Zhang C. Zhao Y. (2013). Incentive problems in china’s new rural pension program. *Labor Market Issues China.* 37 181–201. 10.1108/S0147-912120130000037010

[B29] Lusardi A. (2019). Financial literacy and the need for financial education: evidence and implications. *Swiss. J. Econ. Stat* 155 1–8. 10.1186/s41937-019-0027-5

[B30] Lusardi A. Mitchell O. S. (2010). Implications for retirement wellbeing of financial literacy and planning. *SSRN Elec. J.* 2248:18. 10.2139/ssrn.1695146

[B31] Lusardi A. Mitchell O. S. (2014). The economic importance of financial literacy: theory and evidence. *J. Econ. Lit.* 52 5–44. 10.1257/jel.52.1.5 28579637 PMC5450829

[B32] Lusardi A. Mitchelli O. S. (2007). Financial literacy and retirement preparedness: Evidence and implications for financial education. *Bus. Econ.* 42 35–44. 10.2145/20070104 20070104

[B33] Mahendru M. (2020). Financial well-being for a sustainable society: a road less travelled. *Q. Res. Organ. Manag.* 16 572–593. 10.1108/QROM-03-2020-1910

[B34] Oliver A. G. Campbell R. Graffin S. Bundy J. (2022). Media coverage of earnings announcements: How newsworthiness shapes media volume and tone. *J. Manag.* 10.1177/01492063221080125 [Epub ahead of print].

[B35] Ouimet P. Tate G. (2020). Learning from coworkers: Peer effects on individual investment decisions. *J. Financ.* 75 133–172. 10.1111/jofi.12830

[B36] Robb C. A. Woodyard A. (2011). Financial knowledge and best practice behavior. *J. Financ. Couns. Plann* 22 60–70.

[B37] Rostamkalaei A. Nitani M. Riding A. (2019). Self-employment, financial knowledge, and retirement planning. *J. Small. Bus. Manage.* 60 63–92. 10.1080/00472778.2019.1695497

[B38] Sayinzoga A. Bulte E. H. Lensink R. (2016). Financial literacy and financial behaviour: experimental evidence from rural Rwanda. *Econ. J.* 126 1571–1599. 10.1111/ecoj.12217

[B39] Sconti A. (2022). Digital vs. in-person financial education: What works best for Generation Z? *J. Econ. Behav. Organ.* 194 300–318. 10.1016/j.jebo.2021.12.001

[B40] Semadeni M. Withers M. C. Trevis Certo S. (2014). The perils of endogeneity and instrumental variables in strategy research: Understanding through simulations. *Strategic. Manage. J.* 35 1070–1079. 10.1002/smj.2136

[B41] Shaton M. (2017). The display of information and household investment behavior. *Financ. Econ. Discuss. Ser* 2017:69. 10.17016/feds.2017.043

[B42] Shu L. (2018). The effect of the new rural social pension insurance program on the retirement Shu, L. (2018). The effect of the new rural social pension insurance program on the retirement and labor supply decision in China. *J. Econ. Ageing.* 12 135–150. 10.1016/j.jeoa.2018.03.007

[B43] Simon H. A. (1957). *Administrative Behavior:A Study of DecisionMaking Process in Administrative Organization.* London: Macmillan Education.

[B44] Song C. (2020). Financial illiteracy and pension contributions: a field experiment on compound interest in China. *Rev. Financ. Stud.* 33 916–949. 10.1093/rfs/hhz074

[B45] Stolper O. A. Walter A. (2017). Financial literacy, financial advice, and financial behavior. *J. Bus. Econ.* 87 581–643. 10.1007/s11573-017-0853-9

[B46] Struckell E. M. Patel P. C. Ojha D. Oghazi P. (2022). Financial literacy and self employment– The moderating effect of gender and race. *J. Bus. Res.* 139 639–653. 10.1016/j.jbusres.2021.10.003

[B47] Tang L. Sun S. Yang W. (2021). Investments in human capital: The evidence from China’s new rural pension scheme. *Res. Int. Bus. Financ.* 55:101345. 10.1016/j.ribaf.2020.101345

[B48] Thurstone L. L. (1931). Multiple factor analysis. *Psychol. Rev.* 38 406–427. 10.1037/h0069792

[B49] Tian G. N. Zhou S. Y. Hsu S. (2020). Executive financial literacy and firm innovation in China. *Pac-Basin. Financ. J.* 62 101348. 10.1016/j.pacfin.2020.101348

[B50] van Rooij M. C. J. Lusardi A. Alessie R. J. M. (2012). Financial literacy, retirement planning and household wealth. *Econ. J.* 122 449–478. 10.1111/j.1468-0297.2012.02501.x

[B51] van Rooij M. Lusardi A. Alessie R. (2011). Financial literacy and stock market participation. *J. Financ. Econ.* 101 449–472. 10.1016/j.jfineco.2011.03.006

[B52] Wang J. Song X. (2022). The effect of limited attention and risk attitude on left-tail reversal: Empirical results from a-share data in China. *Financ. Res. Lett* 46:102089. 10.1016/j.frl.2021.102089

[B53] Wiersema M. F. Bowen H. P. (2009). The use of limited dependent variable techniques in strategy research: issues and methods. *Strategic. Manag. J.* 30 679–692. 10.1002/smj.758

[B54] Xiao J. J. Porto N. (2017). Financial education and financial satisfaction. *Int. J. Bank. Mark.* 35 805–817. 10.1108/ijbm-01-2016-0009

[B55] Xu N. Shi J. Rong Z. Yuan Y. (2020). Financial literacy and formal credit accessibility: evidence from informal businesses in China. *Financ. Res. Lett* 36:101327. 10.1016/j.frl.2019.101327

[B56] Xu S. Yang Z. Tong Z. Li Y. (2021). Knowledge changes fate: Can financial literacy advance poverty reduction in rural households? Singap. *Econ. Rev* 38 1–36. 10.1142/s0217590821440057

[B57] Yang T. Y. Tsai P. H. Chiang T. F. (2022). The effect of financial knowledge on asset allocation for Chinese households. *Pac. Econ. Rev* 10.1111/1468-0106.12390 [Epub ahead of print].

[B58] Yang Z. Ali S. T. Ali F. Sarwar Z. Khan M. A. (2020). Outward foreign direct investment and corporate green innovation: an institutional pressure perspective. *S. Afr. J. Bus. Manag.* 51 91–102. 10.4102/sajbm.v51i1.1883

[B59] Yoong J. (2010). *Financial Illiteracy and Stock Market Participation: Evidence From the Rand American Life Panel. Ssrn: Pension Research Council Working Paper.* Philadelphia, PA: University of Pennsylvania.

[B60] Zhang T. (2019). Which policy is more effective, carbon reduction in all industries or in high energy-consuming Industries?–From dual perspectives of welfare effects and economic effects. *J. Clean. Prod.* 216 184–196. 10.1016/j.jclepro.2019.01.183

[B61] Zhao C. Qu X. (2021). Peer effects in pension decision-making: evidence from China’s new rural pension scheme. *Labour. Econ* 69:101978. 10.1016/j.labeco.2021.101978

[B62] Zhao Q. Brosig S. Luo R. Zhang L. Yue A. Rozelle S. (2016). The new rural social pension program in rural China: participation and its correlates. *China. Agr. Econ. Rev.* 8 647–661. 10.1108/caer-07-2016-0116

[B63] Zou J. Deng X. (2019). Financial literacy, housing value and household financial market participation: evidence from urban China. *China. Econ. Rev.* 55 52–66. 10.1016/j.chieco.2019.03.008

