# Supplementary material for: Does Financial Literacy Affect Household Financial Behavior? The Role of Limited Attention
Source: Front Psychol. 2022 Jun 20;13:906153. doi: 10.3389/fpsyg.2022.906153 (PMC9252460; doi:10.3389/fpsyg.2022.906153)
Supplement: Supplementary file 1 [file Table_1.docx]

**Appendix**

**TABLE S1** | Definitions and descriptive statistics of primary variables

| Variable | Symbol | N | Mean | Standard Deviation | Median | Min | Max | Definitions |
| --- | --- | --- | --- | --- | --- | --- | --- | --- |
| Financial literacy | *FL* | 20577 | 1.4143 | 0.8315 | 1 | 0 | 3 | It is calculated by adding up scores in correctly answered interest rate calculations, inflation, and venture capital questions using the CHFS data in 2015. |
| Limited attention | *LA* | 20577 | 0.4347 | 0.4957 | 0 | 0 | 1 | It is a dummy variable that takes 1 if the respondent is always concerned or often concerned about economic and financial information. |
| Holding formal bank accounts | *BA* | 20577 | 0.8191 | 0.3849 | 1 | 0 | 1 | It is a dummy variable that takes 1 if the respondent answered that he or she holds a RMB current deposit account or holds an unexpired RMB time deposit, otherwise 0. |
| Involvement in stock market | *SM* | 20577 | 0.1636 | 0.3700 | 0 | 0 | 1 | It is a dummy variable that takes 1 if the respondent answers yes, otherwise 0. |
| Involvement in venture market | *VM* | 20577 | 0.1970 | 0.3977 | 0 | 0 | 1 | It is a dummy variable that takes 1 if the respondent answers that he or she holds one of the financial accounts; it means that the value of participating in the risky financial market is assigned a value of 1, otherwise 0. |
| Social medical insurance | *SMI* | 20577 | 0.9235 | 0.2659 | 1 | 0 | 1 | It is a dummy variable that takes 1 if the respondent has social medical insurance, otherwise 0. |
| Commercial insurance | *CHI* | 20577 | 0.1183 | 0.3230 | 0 | 0 | 1 | It is a dummy variable that takes 0 if the respondent answers none, which means that he or she does not have commercial insurance, otherwise 1. |
| Pension plans | *PP* | 20577 | 0.8010 | 0.3992 | 1 | 0 | 1 | It is a dummy variable that takes 0 if the respondent answers none, which means that the respondent has no social pension insurance, otherwise 1. |
| Holding credit cards | *CC* | 20577 | 0.2707 | 0.4443 | 0 | 0 | 1 | It is a dummy variable that takes 1 if the respondent answers yes, otherwise 0. |
| Gender | *GENDER* | 20577 | 0.7280 | 0.4450 | 1 | 0 | 1 | It is a dummy variable that takes 1 if the head of the household gender is male, otherwise 0. |
| Age | *AGE* | 20577 | 50.3707 | 14.3211 | 50 | 17 | 96 | We use 2015 minus the year of birth of the head of household. |
| Age ^2^ | *AGE2* | 20577 | 274.59 | 1505.977 | 2500 | 289 | 9216 | It is the square of use 2015 minus the year of birth of the head of household. |
| Education | *EDU* | 20577 | 3.9661 | 1.7670 | 3 | 1 | 9 | We assigned a value of 1-9 in order from “no school, elementary school, junior high school, high school, technical secondary school/vocational high school, college/high vocational school, undergraduate, master’s degree, and doctoral degree.” |
| Married | *MAR* | 20577 | 0.8698 | 0.3365 | 1 | 0 | 1 | It is a dummy variable that takes 1 if the household head is married or living together, otherwise 0. |
| Health | *HEALTH* | 20577 | 0.5036 | 0.5000 | 1 | 0 | 1 | It is a dummy variable that takes 1 if the head of the household’s health status is good, otherwise 0. |
| Worked | *WORK* | 20577 | 0.6616 | 0.4732 | 1 | 0 | 1 | It is a dummy variable that takes 1 if the head of the household had work in 2015, otherwise 0. |
| Old number | OLD | 20577 | 0.6670 | 0.8676 | 0 | 0 | 6 | Number of children in the family (Age<15) in 2015. |
| Child number | *CHILG* | 20577 | 0.5156 | 0.7353 | 0 | 0 | 8 | Number of elderly people in the family (Age>60) in 2015. |
| Family size | *FS* | 20577 | 1.9078 | 1.4080 | 1 | 1 | 14 | The total population of the family. |
| Risk_like | *RL* | 20577 | 0.1252 | 0.3310 | 0 | 0 | 1 | It is a dummy variable that takes 1 if the respondent chooses “high-risk, high-return projects” or “slightly higher-risk, slightly higher-return projects.” The respondent is considered to be risk-preferred, and the variable is assigned a value of 1, otherwise 0. |
| Risk_aversion | *RA* | 20577 | 0.6001 | 0.4899 | 1 | 0 | 1 | It is a dummy variable if the respondent chooses “slightly lower risk, slightly lower return project, or “unwilling to take any risk”, the respondent is considered to be risk-averse, and the variable is assigned a value of 1, otherwise 0. |
| Home ownership | *HO* | 20577 | 0.7620 | 0.2653 | 0 | 0 | 1 | It is a dummy variable that takes 1 if the house have free property rights, otherwise 0. |
| Owning two or more houses | *OH* | 20577 | 0.1183 | 0.2879 | 1 | 0 | 1 | It is a dummy variable that takes 1 if the respondents own two or more houses, otherwise 0. |
| Total family asset | *TA* | 20577 | 13.0901 | 1.5527 | 13.242 | 0 | 16.811 | Natural logarithm of total household assets in 2015. |
| Total family income | *TI* | 20577 | 10.4596 | 2.2987 | 10.968 | 0 | 15.425 | Natural logarithm of total household income in 2015. |

Note: This table reports the descriptive statistics of the sample of CHFS in 2015.
